# The Slr protein of *Streptococcus pyogenes* is selectively expressed in vivo and is a target for protective antibodies

**DOI:** 10.1038/s44321-026-00470-0

**Published:** 2026-06-15

**Authors:** Matevž Rumpret, Jonas Lannergård, Bodil M Kristensen, Nicola N Lynskey, Connor Bowen, Eskil Johnsson, Olof R Nilsson, Anna Norrby-Teglund, Margaretha Stålhammar-Carlemalm, Gunnar Lindahl, Alex J McCarthy

**Affiliations:** 1 Centre for Bacterial Resistance Biology, Department of Infectious Disease, Imperial College London, London, UK; 2 Division of Medical Microbiology, Department of Laboratory Medicine, Lund University, Lund, Sweden; 3 Department of Veterinary and Animal Sciences, University of Copenhagen, Frederiksberg C, Frederiksberg, Denmark; 4 The Roslin Institute, University of Edinburgh, Easter Bush Campus, Midlothian Scotland, UK; 5 Center for Infectious Medicine, Karolinska Institutet, Stockholm, Sweden; 6 Division of Applied Microbiology, Faculty of Engineering, Lund University, Lund, Sweden

**Keywords:** Immunology, Microbiology, Virology & Host Pathogen Interaction

## Abstract

Vaccine candidates are typically identified through characterization of microbial surface antigens expressed under laboratory conditions. Here, we studied the major human pathogen *Streptococcus pyogenes* and showed that Slr, a lipoprotein encoded by all strains, is not detected on the bacterial surface during growth in broth but nevertheless is targeted by protective antibodies in vivo, as demonstrated by passive and active immunizations in a mouse model of invasive infection. The expression of Slr is governed by the zinc-controlled regulator AdcR, indicating that zinc depletion triggers surface expression of Slr in vivo. During infection in humans and mice, the antibody response to Slr is comparable to that elicited by the classical M protein and is, intriguingly, directed almost exclusively against a region with histidine triad (HT) motifs, an outcome that may represent a mechanism of immune escape. For vaccine development, these data focus interest on Slr and other microbial surface proteins selectively expressed in vivo.

## Synopsis

Vaccine candidates are commonly identified through screening of pathogens cultured under laboratory conditions. We present evidence that the lipoprotein Slr of *Streptococcus pyogenes* is absent from bacteria cultured in broth but is upregulated in vivo and is a promising vaccine candidate.

- Surface expression of Slr is governed by the zinc-controlled regulator AdcR, providing an explanation for repression in laboratory broth and upregulation in vivo.
- Immunization and challenge studies in a mouse model show that antibodies to Slr protect against *S. pyogenes* strains of several M types.
- While Slr has HT and LRR regions of similar size, antibodies elicited in humans and mice are mainly directed against the HT region, implying that the LRR region is subdominant.
- The dominating antibody response to the HT region may represent a mechanism of immune escape.

## Abstract (web summary)

Vaccine candidates are commonly identified through screening of pathogens cultured under laboratory conditions. We present evidence that the lipoprotein Slr of *Streptococcus pyogenes* is absent from bacteria cultured in laboratory broth but is upregulated in vivo and is a promising vaccine candidate.

## Graphical abstract

**Figure:**
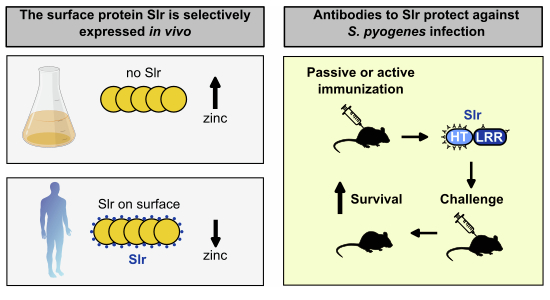

**Figure:**
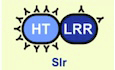

The paper explained **Problem**

*Streptococcus pyogenes* (Group A Streptococcus; GAS) causes common human diseases, such as pharyngitis and skin infections, and also causes life-threatening invasive infections and post-infectious sequelae. Given the importance of *S. pyogenes* as a pathogen, the development of a vaccine has been defined as a priority by the World Health Organization.

**Results**

In this study, we show that antibodies to the conserved *S. pyogenes* lipoprotein Slr protect against experimental infection, making Slr of considerable interest for vaccine development. Notably, Slr is not expressed under standard laboratory conditions but is upregulated during an infection.

**Impact**

While work on vaccine candidates typically is focused on microbes cultivated in the laboratory, our findings focus interest on surface proteins selectively expressed in vivo.

## Introduction

The human pathogen *Streptococcus pyogenes* (Group A *Streptococcus* or GAS) causes a variety of acute diseases, including life-threatening invasive infections and superficial infections of the skin and pharynx. Moreover, an *S. pyogenes* infection may be followed by serious long-term sequelae, such as rheumatic heart disease (Brouwer et al, 2023; Carapetis et al, 2005). The importance of *S. pyogenes* as a pathogen is emphasized by a 2005 estimate that it globally causes more than 500,000 deaths and more than 500 million superficial infections annually (Carapetis et al, 2005). In addition, the societal costs are large because *S. pyogenes* infections occur across the entire age spectrum and cause a highly diverse range of acute and chronic diseases (Cannon et al, 2018; Pfoh et al, 2008). Of further concern is the recent emergence of *S. pyogenes* strains with increased disease-causing capability and the emergence of antibiotic-resistant strains (Barnett et al, 2018). A safe vaccine is therefore required (Good, 2020; Walkinshaw et al, 2023), as underlined by the listing of *S. pyogenes* as a bacterial priority pathogen by the World Health Organization (Sati et al, 2025).

Among potential *S. pyogenes* vaccine candidates, much interest has been focused on the surface-localized M protein, which plays a key role in pathogenesis and is a target for protective antibodies (Bessen et al, 2018; Fischetti, 1989; Harbison-Price et al, 2022; Henningham et al, 2013; Lannergård et al, 2011; Pastural et al, 2020). However, M proteins show extreme variation in sequence among strains, and there is some evidence that antibodies to M protein may play a role in causing rheumatic heart disease (Cunningham, 2019; Gray et al, 2017; Sheel et al, 2016). This situation has motivated extensive studies of other *S. pyogenes* antigens, which potentially may be employed to develop a safe and broadly protective vaccine (Bensi et al, 2012; Fritzer et al, 2010; Harbison-Price et al, 2022; Henningham et al, 2013; Mortensen et al, 2016; Reglinski et al, 2016; Rivera-Hernandez et al, 2016; Rivera-Hernandez et al, 2020; Rodr**í**guez-Ortega et al, 2006; Sanduja et al, 2020; Walkinshaw et al, 2023). Such studies are of interest not only for vaccine development, but also for the molecular insights they may provide about *S. pyogenes* infections and their sequelae.

**Figure 1:**
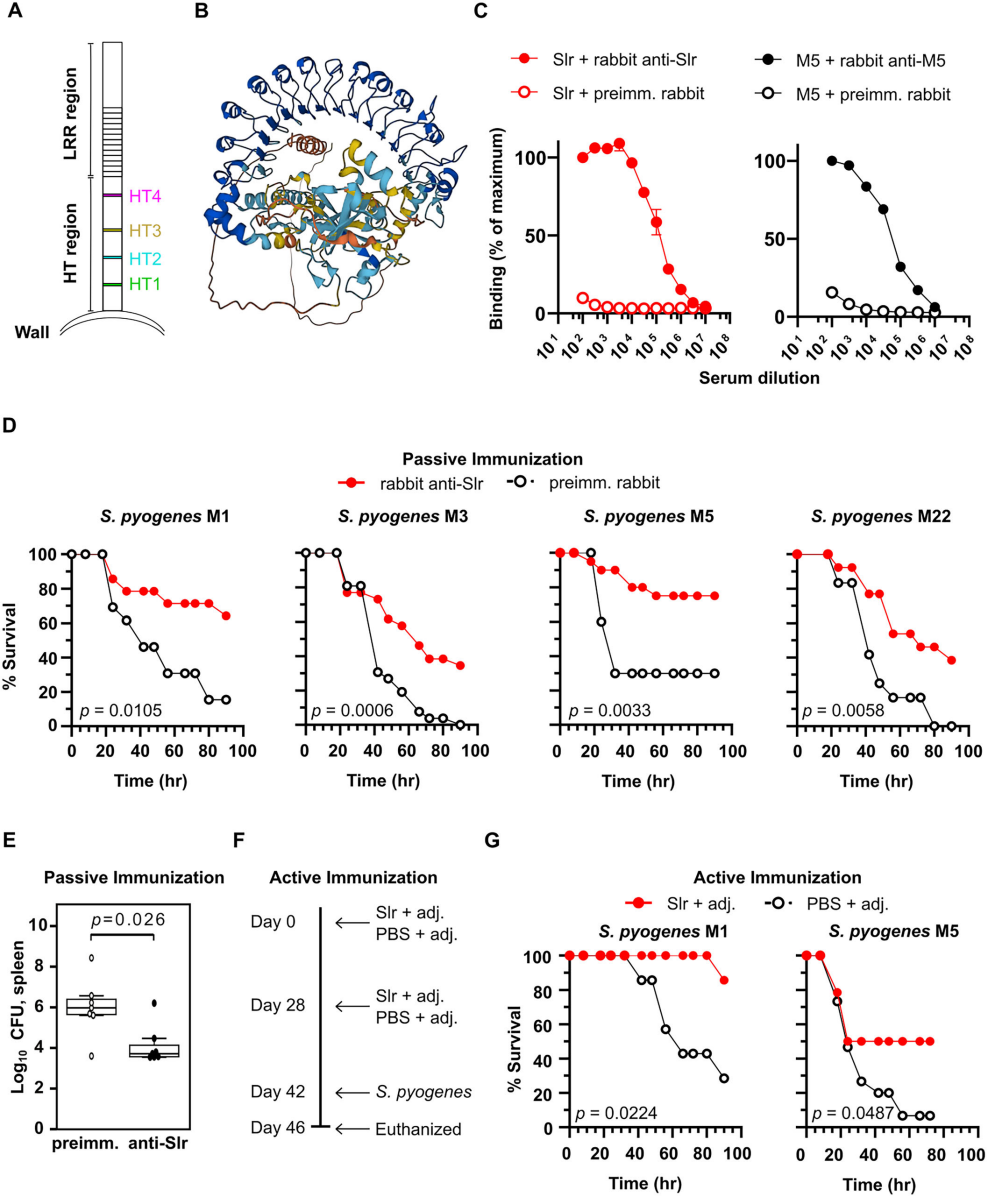
The Slr protein is a target for protective antibodies. (**A**) Schematic of the Slr lipoprotein of *S. pyogenes*. The Histidine Triad (HT) region, which is composed of 4 domains (HT; HT1, HT2, HT3, and HT4), and the Leucine-Rich Repeat (LRR) region, which contains 12 repeats, are indicated. In strain M5 Manfredo, the processed form of Slr has a length of 770 amino acid residues. (**B**) AlphaFold prediction for Slr (Accession: AF-Q99Z76-F1), colored by very high confidence (pLDDT > 90) = blue, high confidence (90 > pLDDT > 70) = cyan, low confidence (70 > pLDDT > 50) = yellow, and very low confidence (pLDDT <50) = orange. (**C**) Antibody reactivity of rabbit antisera raised against the pure Slr and M5 proteins. The sera, and pre-immune serum, were diluted as indicated and tested for reactivity against pure Slr or M5 immobilized in microtiter wells. Mean ± SD from *n* = 3 technical replicates for each serum. (**D**) Passive immunization with rabbit anti-Slr protects mice from systemic *S. pyogenes* infection. Mice were injected by intraperitoneal (i.p.) route with rabbit anti-Slr (red symbols) or pre-immune serum (open symbols) and challenged i.p. with an ~LD_90_ dose of the *S. pyogenes* strain indicated (M1 strain 5448, M3 strain MGAS315, M5 strain Manfredo, or M22 strain AL168). Survival was recorded during a 4-day period. The number of mice used for anti-Slr and the pre-immune control were for M1: *n* = 14 and 13; for M3: *n* = 26 and 26; for M5: *n* = 20 and 20; for M22: *n* = 13 and 12. For the M3 and M5 strains, data are pooled from two separate experiments. (**E**) Passive immunization with anti-Slr reduces *S. pyogenes* growth in the spleens of infected mice. Mice (*n* = 7) were injected i.p. with rabbit anti-Slr or pre-immune serum and challenged i.p. with a sublethal dose of strain M5 Manfredo. The presence of bacteria in the spleens was determined after 20 h. Box-and-whisker plots represent the median, interquartile range (IQR), and non-extreme minimum and maximum values, defined as datapoints falling within 1.5 × IQR from the first and third quartiles. Statistical significance was determined using the unpaired two-tailed Student’s *t* test. (**F**) Schedule for active immunization with Slr. Groups of mice were immunized by subcutaneous (s.c.) route on days 0 and 28 with either 20 µg Slr formulated with adjuvant (CAF01) or with PBS and adjuvant, challenged i.p. with *S. pyogenes* on day 42, and monitored for a 4-day period. (**G**) Survival of actively immunized mice after challenge with an ~LD_90_ dose of strain 5448 (M1) or strain Manfredo (M5). The number of mice used for immunization with Slr and for controls was: M1 infection, *n* = 7 and 7; M5 infection, *n* = 14 and 15. For the passive and active immunizations, the results are displayed as Kaplan–Meier survival curves, analyzed by the log-rank test. All specific *P* values are available in Table EV3. Source data are available online for this figure.

Here, we describe studies of the *S. pyogenes* protein Slr, initially identified as a putative lipoprotein, in which one region includes four short histidine triad (HT) motifs, while another region has leucine-rich repeats (LRRs) (Fig. 1A) (Panina et al, 2003; Reid et al, 2003; Sutcliffe and Harrington, 2002). While regions with HT motifs have been implicated in zinc uptake in streptococci (Adamou et al, 2001; Bersch et al, 2013; Luo et al, 2018; Makthal et al, 2020; Plumptre et al, 2012; Riboldi-Tunnicliffe et al, 2005; Turner et al, 2017), regions with LRRs commonly promote protein-protein interactions (Bella et al, 2008). This is exemplified by eukaryotic pattern-recognition receptors (Botos et al, 2011) and by internalin A (InlA) of *Listeria monocytogenes* (Bonazzi et al, 2009). Accordingly, the presence of both HTs and LRRs in Slr makes it of particular interest to characterize this protein, which contributes to *S. pyogenes* virulence in animal models (Kachroo et al, 2020; Reid et al, 2003). Studies of Slr are also of relevance because the gene for a related protein has been identified in other species of pyogenic streptococci (Plumptre et al, 2012; Shao et al, 2013; Waldemarsson et al, 2006). Thus, Slr is the founding member of a family of streptococcal proteins with HTs and LRRs, implying that studies of Slr will provide insights of broad interest.

While the properties of Slr suggest that it could be a target for protective immunity, immunization with Slr (Spy1361) yielded negative or inconclusive results in two studies focused on the identification of novel *S. pyogenes* vaccine candidates (Bensi et al, 2012; Fritzer et al, 2010). Moreover, it has remained unclear whether Slr, which was reported to be abundantly exposed on the surface of bacteria cultivated in the laboratory (Reid et al, 2003), is exposed more than marginally (Bensi et al, 2012; Waldemarsson et al, 2006). Insights about these problems are provided by the data presented here, which show that Slr is virtually absent from the surface of *S. pyogenes* strains cultivated in broth but nevertheless is a target for protective antibodies in infected mice, as demonstrated in passive and active immunization experiments with several strains. These and other data indicate that formation of Slr is turned on in vivo, making it a target for antibodies. Intriguingly, analysis of the human antibody response to Slr showed that the HT region is immunodominant, while the LRR region is subdominant, a finding of relevance to vaccine development (Lindahl, 2020; Stalhammar-Carlemalm et al, 2007). Together, our data focus interest on Slr as a vaccine candidate with unique structural features and support the notion that a target for protective immunity is not necessarily expressed under standard laboratory conditions, but may be selectively expressed in vivo (Chiang et al, 1999; Mahan et al, 1993b; Smith, 1958).

## Results

### Protection of mice against systemic *S. pyogenes* infection by antibodies to Slr

**Figure EV1:**
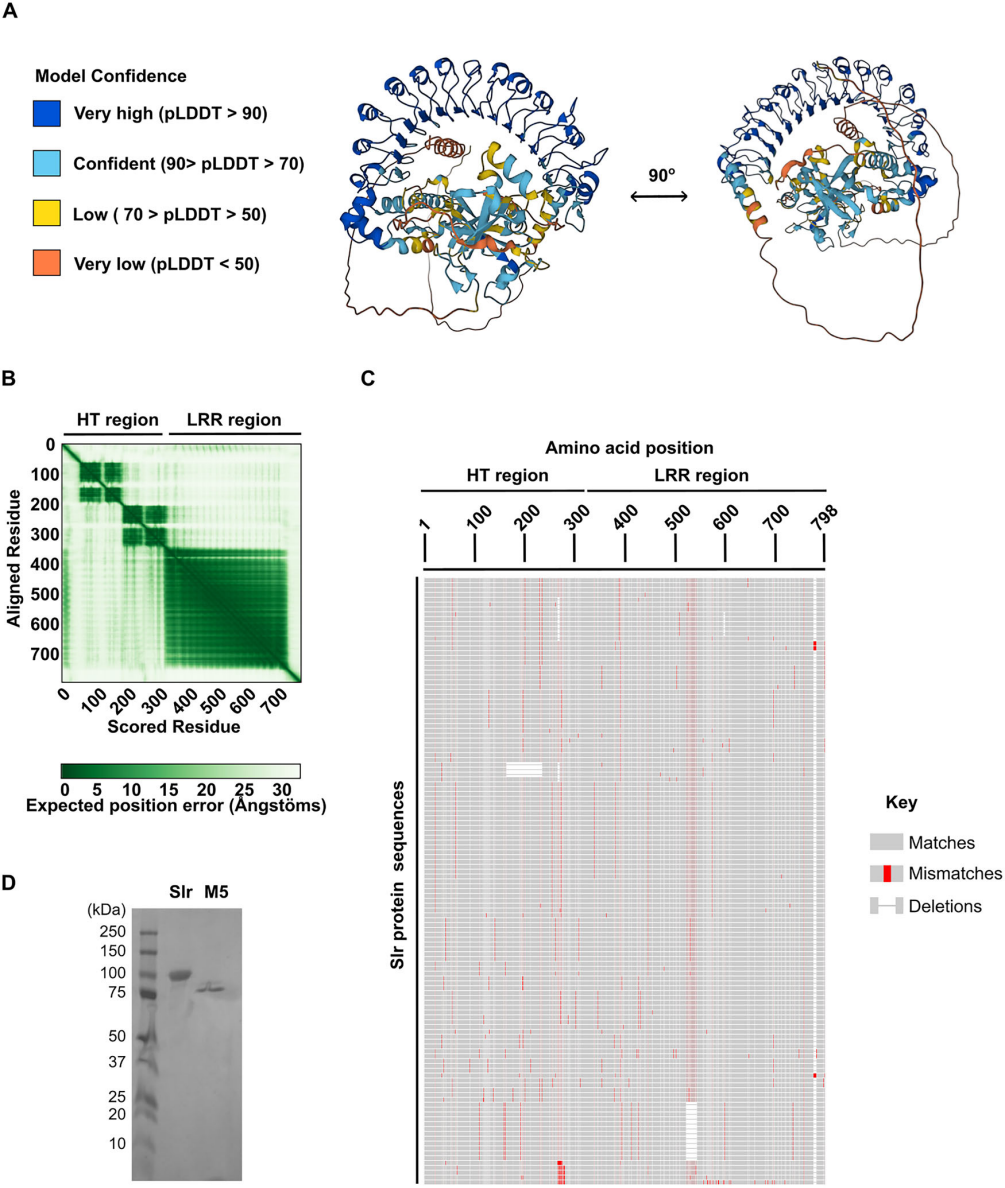
Structure of the Slr protein and sequence conservation. (**A**) AlphaFold prediction for Slr (Accession: AF-Q99Z76-F1), shown in two orientations. The prediction is colored by the model confidence band. (**B**) The expected positional error panel of the predicted Slr structure shows dark green patches for the four histidine triad (HT) domains and leucine repeat region (LRR), indicating that they are well-defined. Note that the green shading is weak between the four HT domains and between the HT and LRR regions, indicating that the inter-domain configuration is not well-defined. (**C**) Multiple sequence analysis of Slr from 125 *S. pyogenes* genomes on PanX software package, visualized using NCBI MSA Viewer 1.25.3. Each horizontal line represents one Slr protein sequence. Variation in sequence is represented by red coloring, with the shade of red indicative of frequency. (**D**) Expression and purification of recombinant Slr, compared with pure recombinant M5.

The Slr protein is encoded by the largely conserved *slr* gene, which is found in all *S. pyogenes* strains (Reid et al, 2003; Shao et al, 2013) and is known as *spy1361* in the reference strain SF370 (Ferretti et al, 2001). The AlphaFold structural model indicated that Slr is a globular protein with well-defined tertiary structure (Figs. 1B and  EV1A,B). Multiple sequence alignment of the Slr protein from 125 *S. pyogenes* genomes confirmed sequence conservation (Fig. EV1C). Although previous immunization studies with Slr had yielded negative or inconclusive results (Bensi et al, 2012; Fritzer et al, 2010), we re-investigated this problem, since the conservation of Slr and its role in virulence suggested that it could be of interest as a vaccine candidate. Therefore, we performed passive and active immunization experiments, using a mouse model of invasive *S. pyogenes* infection. The passive immunizations employed rabbit antiserum, raised by immunization with highly purified recombinant Slr, derived from strain M5 Manfredo (Fig. EV1D), and mixed with Freund’s adjuvant. This rabbit anti-Slr had a high Slr-specific antibody titer, as seen in a comparison with rabbit antiserum to the M5 protein (Fig. 1C), which like other M proteins elicits a strong antibody response (Lannergård et al, 2011). We performed passive immunizations by injecting mice with rabbit anti-Slr serum or pre-immune serum, followed by a challenge with an ~LD_90_ dose of M1, M3, M5, or M22 serotype *S. pyogenes*. Protection against lethal *S. pyogenes* infection was in each case provided by rabbit anti-Slr but not by pre-immune control serum (Fig. 1D). Although protection was not complete, it was significant in all cases. As demonstrated for mice infected with *S. pyogenes* M5, passive immunization with rabbit anti-Slr also caused a significantly decreased bacterial load in the spleens of infected mice (Fig. 1E). Next, we investigated if Slr elicited a protective immune response after active immunization. Here, mice were immunized with pure Slr or PBS control, combined with the adjuvant CAF01, on days 0 and 28, and were challenged by infection with *S. pyogenes* serotype M1 or M5 on day 42 (Fig. 1F). In these experiments, the actively immunized mice were protected from lethal infection, as compared to sham-immunized mice (Fig. 1G). Collectively, these data indicate that Slr is a potent immunogen, which elicits antibodies protecting mice against *S. pyogenes* strains of diverse M types.

### Slr is not detected on the *S. pyogenes* surface under standard laboratory conditions

**Figure 2:**
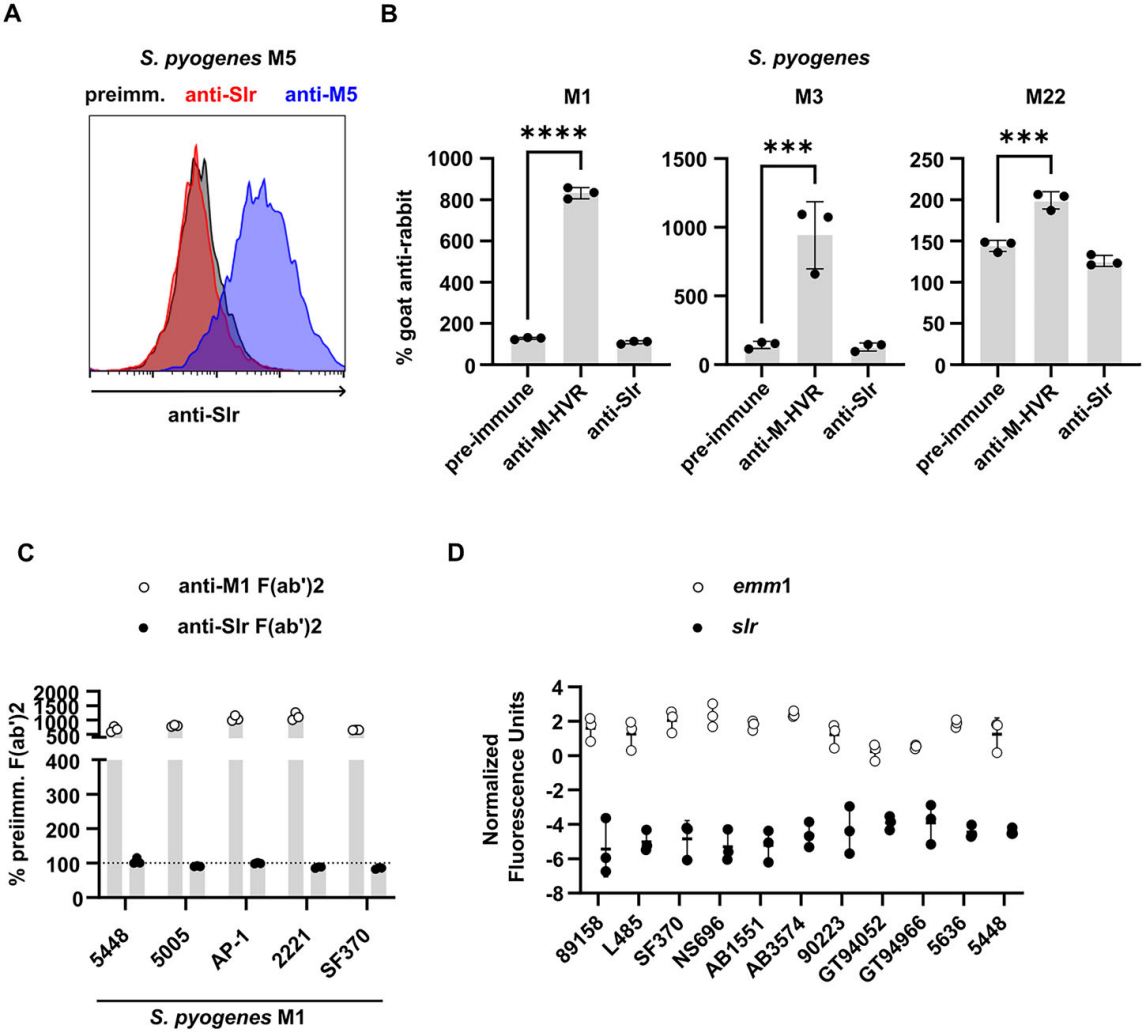
The Slr protein is not detected on the surface of *S. pyogenes* grown in TH-Y broth. (**A**) Surface expression of Slr and M5 protein on strain M5 Manfredo, determined by flow cytometry. Rabbit antisera against Slr and M5 were used, with pre-immune rabbit serum as a control. The data are representative of *n* = 3 biological replicates. (**B**) Expression of Slr and M proteins on the surface of *S. pyogenes* M1, M3, and M22 strains cultured in TH-Y. Flow cytometry analysis of M1 strain SF370, M3 strain 771, and M22 strain AL168, studied with F(ab’)2 fragments of the antibodies indicated. Mean ± SD from  *n* = 3 biological replicates are shown. Ordinary one-way ANOVA followed by Dunnett’s multiple comparisons test. *P < 0.05; **P < 0.01; ***P < 0.001; ****P < 0.0001. (**C**) Expression of the Slr and M1 protein on the surface of different *S. pyogenes* isolates of type M1, cultured in TH-Y. Flow cytometry analysis of M1 strains 5448, MGAS5005 (5005), AP-1, 2221 and SF370. The analysis employed rabbit F(ab’)2 fragments, and the data are presented as % of the signal obtained with F(ab’)2 generated from pre-immune serum. Means ± SD from *n* = 3 biological replicates are shown. (**D**) Mid-logarithmic phase transcriptomic analysis of different M1 isolates cultured in TH-Y as reported by Maamary et al (Maamary et al, 2012b), data ref: GEO Accession GSE35568 (Maamary et al, 2012a), showing expression of the Slr (*slr*) and M1 (*emm1*)-encoding genes in the M1 isolates. Data are presented as normalized fluorescence units. All specific *P* values are available in Table EV3. Source data are available online for this figure.

While our immunization experiments indicated that Slr is a target for protective antibodies, it has, surprisingly, remained unclear whether Slr is exposed on the surface of *S. pyogenes* (Bensi et al, 2012; Bober et al, 2011; Reid et al, 2003; Waldemarsson et al, 2006). To re-investigate this question, we cultivated our reference *S. pyogenes* M5 strain under standard laboratory conditions, i.e., in Todd–Hewitt broth supplemented with yeast extract (TH-Y), and employed rabbit anti-Slr and flow cytometry analysis to study surface expression of Slr. We used the M5 protein and rabbit anti-M5 sera as a control and found clear evidence for surface exposure of the M5 protein, as expected (Fig. 2A). However, when the bacteria were stained with rabbit anti-Slr there was no difference from the pre-immune control, indicating that Slr could not be detected on the bacterial surface (Fig. 2A). Importantly, this result cannot be explained by a low titer for anti-Slr, since this antiserum had a titer that was even higher than that of rabbit anti-M5 sera (Fig. 1C). These results indicated that Slr is not displayed at a detectable level on the surface of the *S. pyogenes* M5 strain growing in standard laboratory medium.

**Figure EV2:**
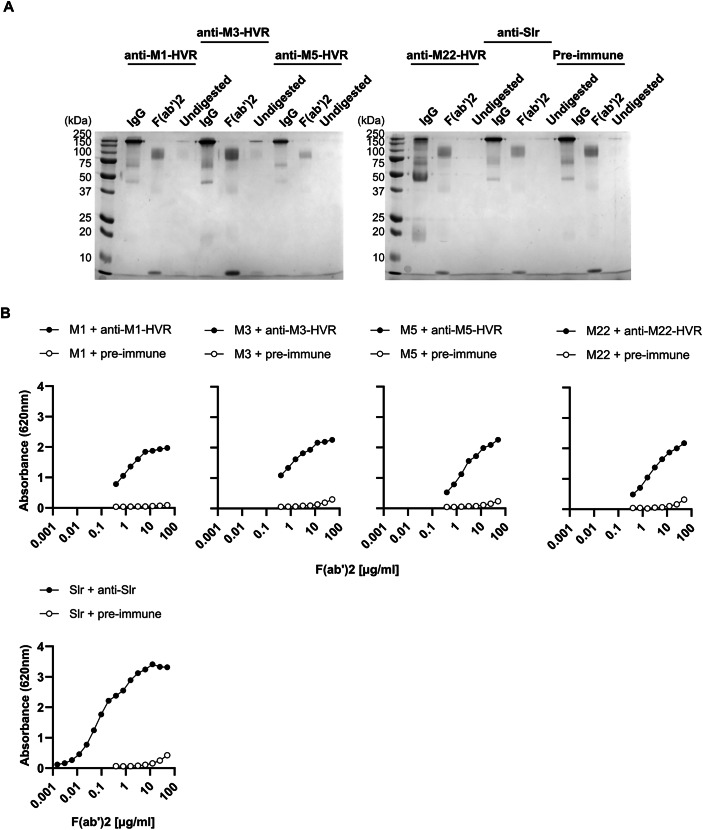
Purification and reactivity of F(ab’)2 fragments derived from anti-M-HVR and anti-Slr. (**A**) IgG was purified from rabbit antisera (raised against M1-HVR, M3-HVR, M5-HVR, M22-HVR, or against Slr) and from pre-immune rabbit serum, using a protein G column. F(ab’)2 fragments were prepared by digestion of purified IgG with pepsin and purified using protein A columns. Input IgG, F(ab’)2, and the unsuccessfully digested portion of the input IgG were separated by SDS-PAGE analysis. (**B**) To verify that the F(ab’)2 fragments generated from rabbit anti-M (HVR) and anti-Slr antibodies retained reactivity to the corresponding intact M proteins or Slr, they were analyzed, as indicated, for reactivity with the pure M protein or Slr immobilized in microtiter wells. F(ab’)2 fragments derived from pre-immune IgG were used as a control. F(ab’)2 interaction with immobilized proteins was detected by ELISA. Means ± SD from *n* = 3 independent experiments are shown.

In contrast to our results with the M5 strain, previous studies of two *S. pyogenes* M1 strains reported surface expression of Slr on bacteria cultivated in TH-Y (Bober et al, 2011; Reid et al, 2003). To resolve this apparent contradiction, we extended the flow cytometry studies to strains of types M1, M3, and M22 by analyzing surface expression of Slr and the corresponding M protein. Since many *S. pyogenes* strains (but not our reference M5 strain) show reactivity with the Fc part of rabbit IgG, these experiments employed F(ab’)2 fragments (Fig. EV2A), which had been derived from highly specific rabbit IgG antibodies raised against Slr or against the hypervariable region (HVR) of the relevant M protein. These F(ab’)_2_ fragments generated from anti-Slr and anti-HVR rabbit IgG demonstrated the expected reactivity against target antigens, making them suited for analysis by flow cytometry (Fig. EV2B). Binding of the rabbit F(ab’)2 fragments to bacteria was analyzed with goat anti-rabbit IgG because goat IgG lacks Fc-reactivity with *S. pyogenes* proteins (Stenberg et al, 1992). In experiments with the M1, M3 and M22 strains, the M protein was readily detected on the bacterial surface, but the signal obtained with anti-Slr F(ab’)2 fragments was not higher than for the control F(ab’)2 fragments (Fig. 2B). Taken together, our flow cytometry analysis of M1, M3, M5 and M22 strains indicated that Slr is absent from bacteria growing in TH-Y.

Given the reports that Slr is expressed on the surface of two M1 strains cultivated in TH-Y, we extended the flow cytometry analysis to additional M1 strains, including the two isolates (strains 5005 and AP-1) proposed to express Slr on the surface (Bober et al, 2011; Reid et al, 2003). In these experiments, we readily detected the M1 protein on the surface of the five strains tested, but the reactivity with anti-Slr F(ab’)2 fragments was as low as for F(ab’)2 fragments derived from pre-immune rabbit IgG (Fig. 2C). Thus, Slr was not detected on the surface of any of the five M1 strains, confirming that Slr is absent from the surface of *S. pyogenes* cultivated in TH-Y broth.

To further validate the observation that Slr is not expressed during in vitro culture in TH-Y, we examined pre-existing transcriptome microarray data from *S. pyogenes* M1 isolates, which had been analyzed for expression of many genes, including *emm1* and *slr*, after cultivation in TH-Y (Maamary et al, 2012b). Whilst *emm1* expression was pronounced in all 11 *S. pyogenes* M1 isolates, there was exceptionally low abundance of *slr* transcripts (Fig. 2D), supporting our conclusion that Slr is absent from M1 strains and other *S. pyogenes* strains cultivated in TH-Y. In summary, studies performed at the protein level or at the transcript level agree that Slr is not expressed at detectable levels on *S. pyogenes* cultivated under standard laboratory conditions.

### Surface expression of Slr is governed by the Zn-controlled regulator AdcR

**Figure 3:**
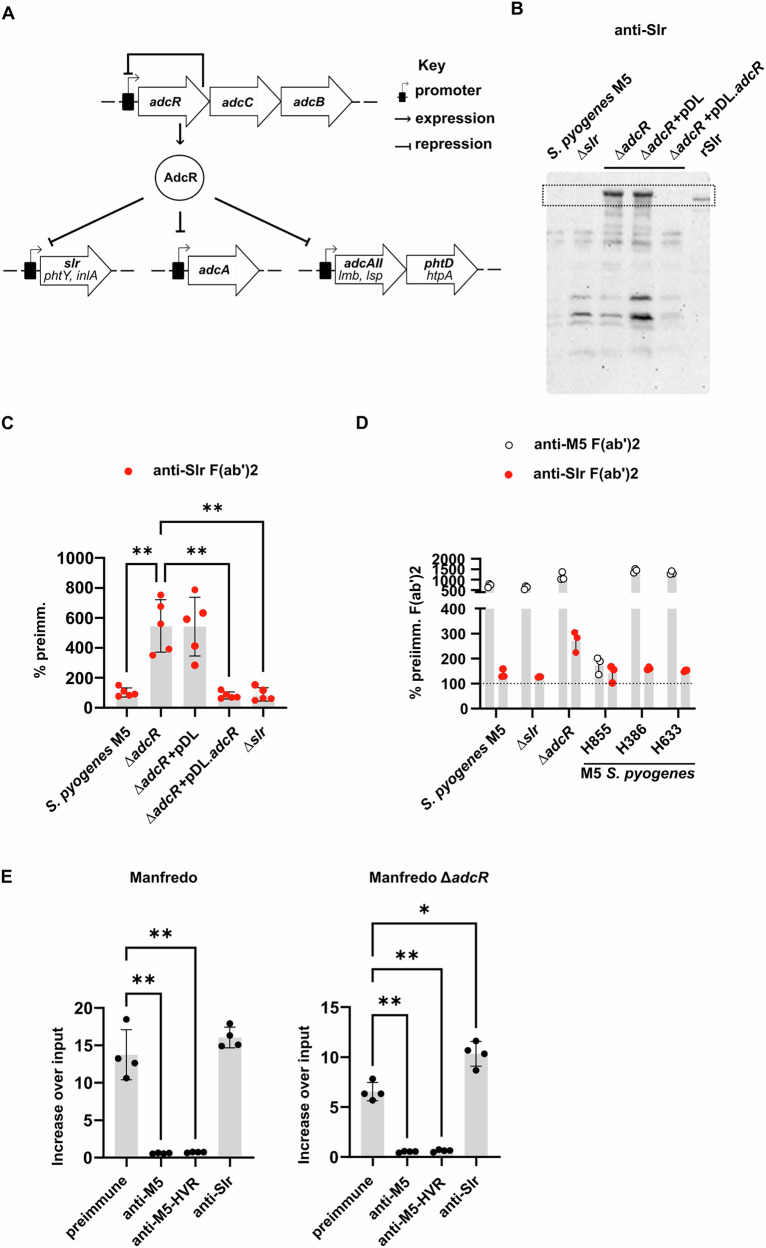
Slr expression is controlled by the global Zn-regulator AdcR. (**A**) *S. pyogenes* genes implicated in Zn uptake (Brenot et al, 2007; Sanson et al, 2015). These genes are part of the AdcR regulon and are upregulated in the absence of Zn, while the presence of Zn causes repression by the global regulator AdcR, which binds to specific operators (shaded boxes). The AdcABC proteins form an ABC transporter, while the AdcAII protein shows similarity to AdcA and contributes to zinc acquisition by unknown mechanisms. The PhtD and Slr proteins have HT motifs that may allow binding of environmental zinc, for subsequent delivery to transporters (Makthal and Kumaraswami, 2017). While the PhtD protein contributes to zinc acquisition in *S. pyogenes*, the situation is unclear for Slr (Makthal et al, 2020). Alternative gene nomenclature is shown. (**B**) Total Slr expression quantified by Western blot analysis of bacterial extracts, prepared from isogenic Manfredo strains cultured in TH-Y broth. The extracts were analyzed for the presence of Slr, using rabbit anti-Slr as the probe. Pure recombinant Slr (rSlr) was included as a control. Image representative of three independent experiments. (**C**) Slr surface expression is suppressed by the AdcR regulator in *S. pyogenes*. Flow cytometry analysis of isogenic M5 strains (wildtype, Δ*adcR*, Δ*adcR* + *pDL,* or Δ*adcR* + *pDL.adcR*). Surface localization of Slr was detected by incubation with rabbit anti-Slr F(ab’)2 fragments. Data are presented as % signal compared to *S. pyogenes* stained with rabbit pre-immune serum. Means ± SD from *n* = 5 biological replicates are shown. Repeated measures one-way ANOVA followed by Dunnett’s multiple comparisons test. *P < 0.05; **P < 0.01; ***P < 0.001; ****P < 0.0001. (**D**) Expression of Slr and M5 protein on the surface of *S. pyogenes* serotype M5 clinical isolates, cultured in TH-Y. Flow cytometric analysis of M5 strains H855, H386, and H633, using F(ab’)2 fragments of rabbit anti-M5 and anti-Slr for detection. Strain M5 Manfredo and its *Δslr* and *ΔadcR* mutants were included as controls. Data are presented as % signal compared to *S. pyogenes* stained with F(ab’)2 generated from pre-immune serum. Means ± SD from *n* = 3 biological replicates are shown. (**E**) Survival of *S. pyogenes* Manfredo and the isogenic *adcR* knockout after three hours of incubation in whole blood. Strains were opsonized with pre-immune, anti-M5, anti-M5-HVR, or anti-Slr rabbit sera and incubated in whole human blood for three hours. Mean ± SD of data obtained with peripheral blood from four donors are shown. Repeated measures one-way ANOVA followed by Dunnett’s multiple comparisons test. *P < 0.05; **P < 0.01; ***P < 0.001; ****P < 0.0001. All specific *P* values are available in Table EV3. Source data are available online for this figure.

The observation, that Slr is not expressed at a detectable level on the *S. pyogenes* surface during standard laboratory conditions, led us to further investigate the mechanisms by which it is regulated. The *slr* gene is part of an *S. pyogenes* regulon governed by AdcR, a global regulator of zinc uptake (Fig. 3A) (Brenot et al, 2007; Sanson et al, 2015). Under high-zinc conditions, AdcR acts as a repressor, suppressing the expression of genes within the regulon, including *slr*, while zinc depletion causes de-repression of the regulon. The regulon encompasses genes encoding an ABC transporter, as well as the genes *phtD* and *slr*, both of which encode HT-motif-containing proteins potentially involved in binding of environmental zinc (Luo et al, 2018; Makthal et al, 2020; Plumptre et al, 2012; Riboldi-Tunnicliffe et al, 2005).

Since previous studies had demonstrated that transcription of the *slr* gene is repressed by AdcR (Brenot et al, 2007; Sanson et al, 2015), we analyzed whether expression of the Slr protein was controlled by AdcR in our system. To this end, we constructed a Δ*adcR* deletion mutant in the *S. pyogenes* M5 strain and compared Slr expression in the mutant and wild-type. The analysis was first focused on whole-cell lysates, which were analyzed for the presence of Slr by Western blot, employing rabbit anti-Slr for detection. In this analysis, Slr was absent from the wild-type, which was as negative as an isogenic Δ*slr* mutant, but Slr was readily detected in the lysate of the Δ*adcR* deletion mutant (Fig. 3B). Importantly, complementation of the Δ*adcR* strain with a plasmid carrying *adcR*, but not with an empty plasmid, significantly reduced Slr expression and restored the wild-type phenotype. These data show that expression of the Slr protein is controlled by AdcR. We then used flow cytometry analysis to assess whether Slr is found on the surface of the Δ*adcR* mutant cultivated in TH-Y. This analysis demonstrated clear expression of Slr on the surface of the Δ*adcR* mutant, but not on the complemented strain, indicating that AdcR plays a key role in controlling surface expression of Slr (Fig. 3C). Collectively, our findings establish that AdcR negatively regulates Slr expression, making expression of Slr undetectable in wild-type *S. pyogenes* strains cultured in TH-Y.

To verify that the reference M5 strain used for this work does not have unique properties, we compared it with three clinical M5 isolates regarding surface expression of the Slr and M5 proteins. Since some clinical M5 isolates bind IgG-Fc (Whatmore and Kehoe, 1994), this analysis was performed with F(ab’)2 fragments. No surface-level expression of Slr was found in any of the isolates (Fig. 3D), while M5 was present on the surface of all strains (although the level was low for one strain). These data indicate that various M5 strains, like *S. pyogenes* strains of other M types, lack Slr on the surface, when cultivated in TH-Y.

**Figure EV3:**
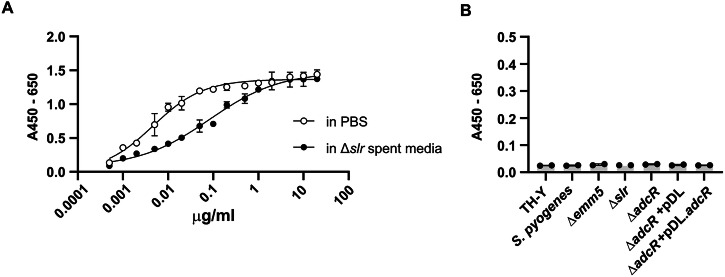
Slr is not detected in *S. pyogenes* culture supernatants. (**A**) ELISA used to detect Slr. Pure Slr was diluted in PBS or in spent TH-Y media (recovered from a culture of the *S. pyogenes* Δ*slr* strain), diluted to the concentrations shown, and coated onto microtiter plates. Reactivity with rabbit anti-Slr IgG was tested. Means ± SD from *n* = 3 technical replicates are shown. (**B**) Detection of Slr in the supernatants of *S. pyogenes* by ELISA. Culture supernatants were coated onto microtiter plates and tested for reactivity with rabbit anti-Slr IgG. Two independent experiments are shown.

While our studies indicated that Slr is not expressed on the surface of wild-type *S. pyogenes* cultivated under standard laboratory conditions, they did not exclude that Slr was secreted into the medium. Although this explanation seemed unlikely, given the very low abundance of *slr* transcripts (Fig. 2D), we directly analyzed whether Slr was present in spent TH-Y medium. A highly sensitive ELISA was developed (Fig. EV3A) and employed to measure Slr in supernatants from the M5 wild-type strain and its Δ*emm5*, Δ*slr*, and Δ*adcR* mutants (Fig. EV3B). In these tests, Slr was not detected in any of the supernatants, i.e., it was absent even for the Δ*adcR* mutant, which expresses Slr on the surface. These results indicate that the absence of Slr from the bacterial surface does not reflect release into the growth medium.

The demonstration that Slr is expressed on the surface of the *adcR* mutant made it possible to initiate an analysis of the role of the functionality of anti-Slr antibodies. A major effector mechanism of antibodies targeting surface-exposed *S. pyogenes* antigens is the facilitation of phagocytic uptake and killing by neutrophils in human whole blood through opsonization. Specifically, we analyzed whether rabbit anti-Slr antibodies, which are protective in a mouse model (Fig. 1), would opsonize the Δ*adcR* mutant for phagocytosis in the classical bactericidal test employing human whole blood (Fig. 3E). As expected, opsonization with either anti-M5 or anti-M5-HVR serum resulted in a significant reduction in survival of Manfredo wild-type and Δ*adcR* strains. In contrast, opsonization with anti-Slr IgG did not reduce outgrowth of Manfredo wild-type, as expected, or Δ*adcR* strains relative to the pre-immune control. These data support the notion that antibodies to Slr primarily act by interfering with Slr function, not by promoting phagocytosis.

### Slr elicits a strong antibody response during *S. pyogenes* infection

**Figure 4:**
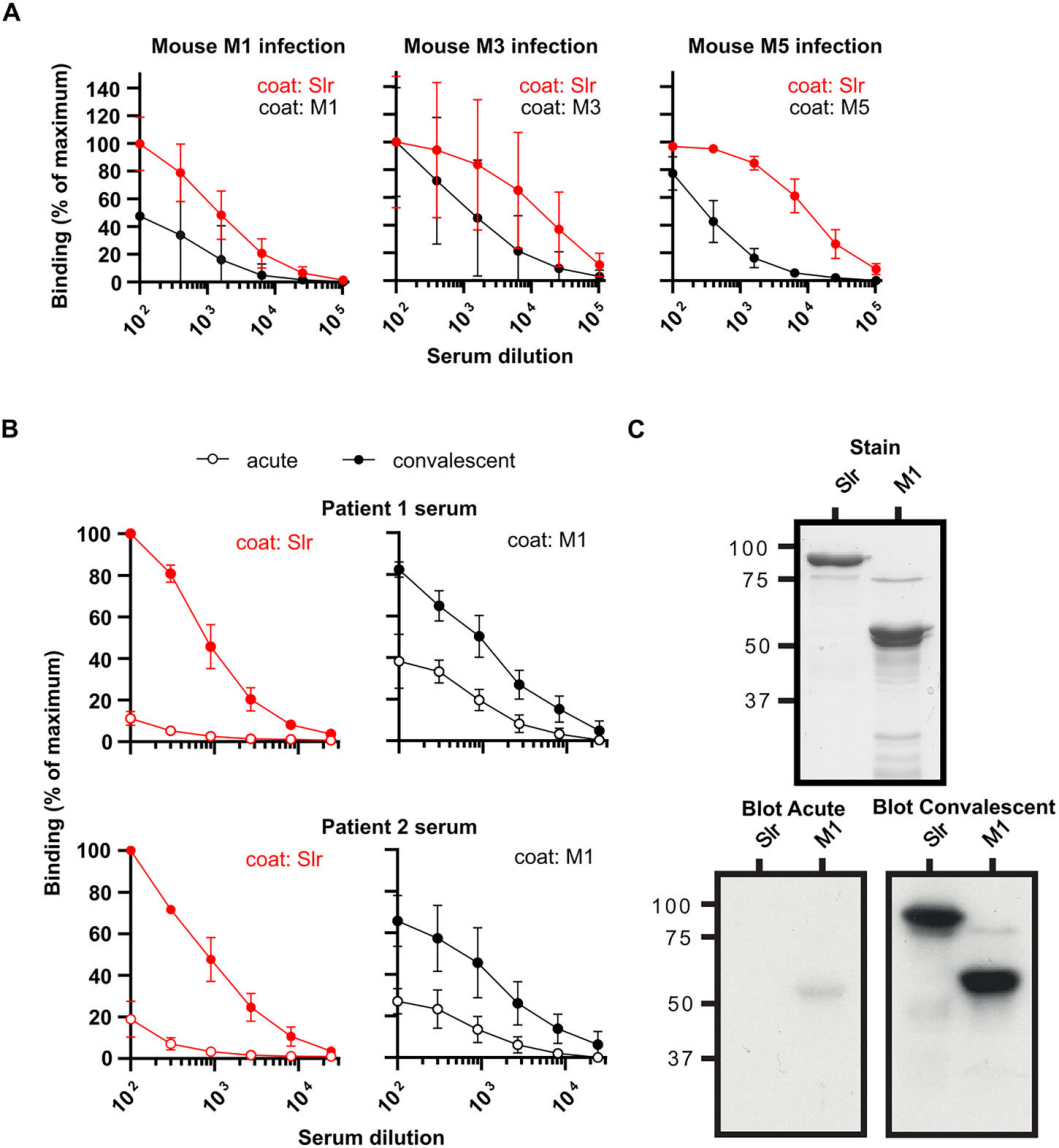
Infection with *S. pyogenes* induces high titers of anti-Slr antibodies. (**A**) Antibody responses in mice. Sera from mice infected by intraperitoneal (i.p.) route with a sublethal dose of *S. pyogenes* M1 (*n* = 6), M3 (*n* = 8), or M5 (*n* = 6) were diluted as indicated and analyzed for reactivity with pure Slr, M1, M3, or M5, respectively, immobilized in equimolar amounts in microtiter wells. Serum from uninfected mice showed no reactivity in these tests. Mean ± SD is shown for each mouse group. (**B**) Antibody responses in humans. Acute and convalescent sera, recovered from two humans with invasive *S. pyogenes* M1 infection, were diluted as indicated and analyzed for reactivity with pure Slr or M1, immobilized in equimolar amounts in microtiter wells. The data for reactivity with M1 are reproduced from (Lannergård et al, 2011), with permission. Mean ± SD of data obtained with *n* = 3 technical replicates is shown for each patient. (**C**) Western blot analysis of the human antibody response. Similar amounts of Slr and M1 were separated by SDS-PAGE, and identical gels were used for transfer to membranes, which were probed as indicated with the sera from patient 1. Bound human antibodies were detected by incubation with radiolabeled protein G and autoradiography. Similar data were obtained for patient 2. Source data are available online for this figure.

The factors that confer immunity to *S. pyogenes* infection remain poorly understood. While it is commonly assumed that M protein elicits type-specific and protective immunity, the role of anti-M antibodies in protection against natural infection remains unclear (Denny et al, 1957; Lannergård et al, 2011). Indeed, recovery from acute infection may be independent of antibodies and T cells (Medina et al, 2001), while protection against prolonged infection may be promoted not only by gradually appearing type-specific antibodies to M protein (Denny et al, 1957; Lannergård et al, 2011), but also by non-type-specific antibodies to conserved antigens (Brouwer et al, 2023; Keeley et al, 2025). Given this situation, and our finding that Slr is a target for protective antibodies in a mouse model (Fig. 1), it was relevant to quantify the antibody response to Slr during infection. We therefore analyzed the response to Slr in *S. pyogenes*-infected mice and humans and compared this response to that elicited by the M protein of the strain. For mice, we analyzed sera recovered 4 weeks after sublethal infection with an M1, M3, or M5 strain and found, remarkably, that the response to Slr was considerably higher than to M protein in all three groups (Fig. 4A), although the bacteria used to infect the mice had been grown in TH-Y and therefore initially lacked Slr on the surface. These data strongly suggest that expression of Slr is turned on during an infection, resulting in an antibody response against Slr. For studies of infected humans, we employed acute and convalescent sera recovered from two patients with invasive M1 infection. Analysis of these sera showed an antibody response to Slr in both patients (Fig. 4B, left), in agreement with previous reports that Slr elicits a response in humans and macaques (Bensi et al, 2012; Reid et al, 2001; Virtaneva et al, 2003). Additionally, there was a clear response to M1 in both patients (Fig. 4B, right), as previously reported (Lannergård et al, 2011). Of note, these studies of patient sera suggested that the responses to Slr and M1 were of comparable strengths. Western blot analysis of acute and convalescent serum from one of the patients confirmed that the infection had elicited a distinct antibody response to both Slr and M1 (Fig. 4C). That the anti-Slr antibodies detected in this analysis indeed were elicited by Slr, and not by a cross-reacting antigen, is strongly supported by the Western blot analysis shown in Fig. 3B, in which lysates prepared from wild-type bacteria and the Δ*adcR* mutant were compared, indicating that anti-Slr mainly, if not exclusively, reacted with Slr. Taken together, the studies of mice and humans indicated that Slr elicits a robust antibody response during infection, but it should be noted that the data did not provide information about the ability of these antibodies to protect against infection.

### In the antibody response to Slr, the LRR region is subdominant

The analysis of antibody responses indicated that Slr elicits a strong response during natural infection. However, it seemed unlikely that this response makes a major contribution to protection, since natural infection with *S. pyogenes* is not known to elicit broad immunity to subsequent infection (Brouwer et al, 2023). We, therefore, hypothesized that most of the antibodies elicited by Slr during an infection lack protective ability, i.e., are directed against non-protective epitopes. This argument focused interest on the concepts of immunodominance and subdominance in antibody responses (Lannergård et al, 2011; Lindahl, 2020). Specifically, studies in several systems have indicated that an immunodominant region, i.e., a region targeted by most antibodies elicited by a protein, may act as a decoy that diverts the response from a subdominant region, which is particularly important as a target for protective antibodies (Lindahl, 2020).

**Figure EV4:**
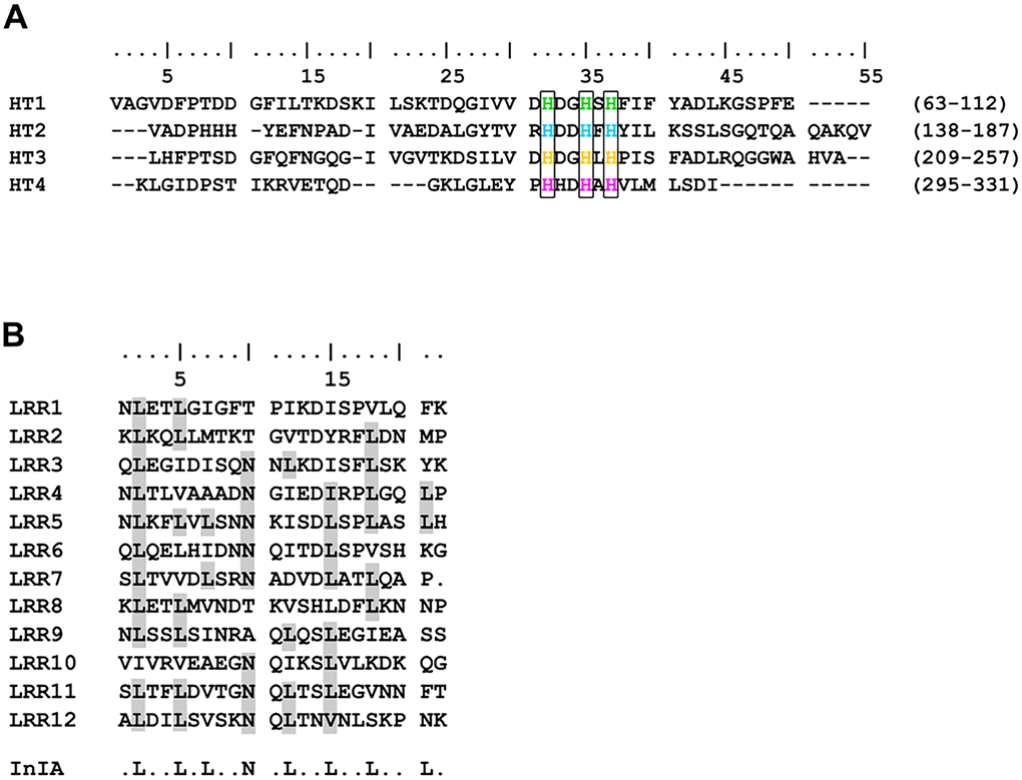
Sequences of repeats in the HT and LRR regions of Slr. (**A**) Alignment of sequences of the Histidine Triad (HT)1, HT2, HT3, and HT4 domains of Slr. The sequences shown are based on the predicted structural features of the HT regions. Each HT is between 37 and 50 amino acid residues long. The Histidine Triad sequence is composed of the consensus sequence HxxHxH. The histidine residues in each HT region are colored. (**B**) Alignment of the 12 repeats in the LRR region. Each LRR has a length of 22 residues, except the seventh one, which is one residue shorter. The consensus sequence of LRRs in InlA of *L. monocytogenes* is indicated in bold below the alignment, and residues at the corresponding positions in Slr are highlighted in gray. Of note, all data in this figure are based on Slr in strain M5 Manfredo.

**Figure 5:**
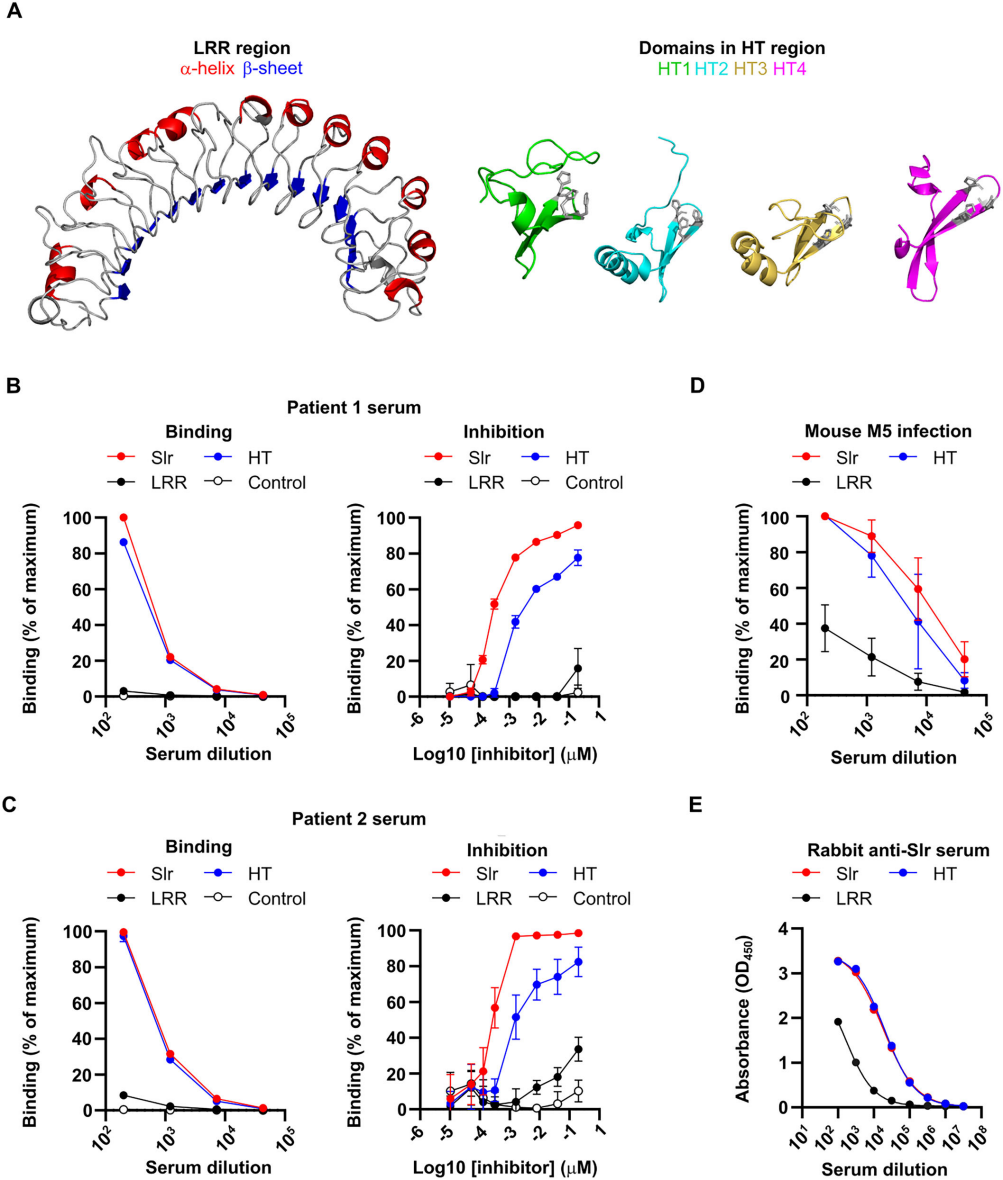
Analysis of the antibody response to Slr: the LRR region is subdominant. (**A**) Structure of the LRR and HT regions of Slr, as predicted from the AlphaFold model of Slr (Accession: AF-Q99Z76-F1). The HT regions are colored in green, cyan, yellow, or magenta for HT1, HT2, HT3, and HT4, respectively, with the histidine residues shown in gray. The LRR region is colored in blue for β-sheets and in red for α-helices. (**B**, **C**) Binding and inhibition tests with anti-Slr antibodies, elicited in two humans with invasive *S. pyogenes* infection. Left panels: binding tests with intact Slr and fragments of Slr comprising the HT or LRR region, with GST protein as control. The proteins were immobilized in equimolar amounts in microtiter wells, and bound antibodies were detected with radiolabeled protein G. Right panels: intact Slr, and the two Slr fragments, were analyzed for ability to inhibit the binding of human anti-Slr antibodies to immobilized Slr. Data are presented as % inhibition. Each panel in (**B**, **C**) shows means ± SD from *n* = 3 technical replicates for each patient. (**D**) Reactivity of mouse antibodies, elicited by sublethal infection with *S. pyogenes* M5 Manfredo, against intact Slr and its HT and LRR fragments. The proteins were immobilized in microtiter wells, and bound antibodies were detected with radiolabelled protein G. Means ± SD from *n* = 11 independently infected mice are shown. (**E**) Reactivity of rabbit anti-Slr, raised with Freund’s adjuvant, with intact Slr and its HT and LRR fragments, immobilized in microtiter wells. IgG deposition was detected by ELISA. Means ± SD from *n* = 3 technical replicates are shown. Source data are available online for this figure.

For Slr, we hypothesized that the antibody response is largely directed against either the HT region or the LRR region (Fig. 1A). The HT region, which is found in the amino-terminal half of Slr and most likely is located proximally to the cell surface, contains four histidine triad motifs that have the consensus amino acid sequence HxxHxH (Fig. EV4A). While little is known about the regions containing HT motifs in Slr, studies of *Streptococcus pneumoniae* proteins have indicated that HT motifs are located within relatively long repeats, which have similar structure but highly divergent amino acid sequences (Bersch et al, 2013; Luo et al, 2018; Riboldi-Tunnicliffe et al, 2005). For Slr, structural analysis similarly indicated the presence of four domains (HT1 to HT4) with highly divergent sequences but similar length and structure (Figs. 5A,  EV1B and EV4A). Regarding the LRR region, it contains 12 LRR motifs, which have conserved residues at positions corresponding to the consensus sequence described for LRRs in *Listeria monocytogenes* InlA (Sabet et al, 2005) (Fig. EV4B). These LRRs are predicted to be stacked into a horseshoe-like solenoid with the β-strands forming the inside-facing β-sheet surface (Fig. 5A). Thus, Slr is mainly composed of two distinct and structurally unrelated repeat regions, the HT region and the LRR region.

To analyze immunodominance and subdominance in the antibody response to Slr, we first employed the two convalescent human serum samples described above, recovered from patients that had responded to Slr (Fig. 4B). In direct binding tests, these sera showed similar reactivity with intact Slr and the isolated HT region, while there was little to no reactivity with the isolated LRR region (Fig. 5B,C). This indicates that the anti-Slr antibodies were directed almost exclusively against the HT region. This conclusion was confirmed by inhibition tests, which showed that binding of the human antibodies to Slr was inhibited almost completely by the HT region, but not by the LRR region (Fig. 5B,C). While the concentration needed for inhibition was higher for the HT region than for intact Slr, these experiments indicate that most anti-Slr antibodies elicited in humans are directed against the HT region. Of note, the almost complete lack of anti-LRR response in the two patients did not reflect expression of an atypical Slr protein in the infecting strains. For both patients, the strain was available and shown to encode an Slr protein with 99% identity to that of the reference strain M5 Manfredo. For analysis of immunodominance in the anti-Slr response elicited by experimental *S. pyogenes* infection, we employed sera recovered from mice infected with a sublethal dose of M5 bacteria (Fig. 4A, right). Analysis of 11 mouse sera demonstrated that they had similar reactivity for full-length Slr and the HT region, while reactivity to the LRR region was considerably lower, in line with the observations in humans (Fig. 5D). Thus, studies of sera from naturally infected humans and experimentally infected mice indicated that the HT region of Slr is immunodominant, while the LRR region is subdominant. Similar results were obtained for the rabbit anti-Slr that had been employed in passive immunization experiments, but for this antiserum the data suggest that the response to the LRR region was more pronounced, while still being subdominant (Fig. 5E). Of note, the strongly immunostimulatory Freund’s adjuvant had been used to raise this rabbit serum, suggesting that it may not recognize the same epitopes as anti-Slr antibodies elicited during an infection. In summary, the antibody response to Slr is directed almost exclusively against the HT region, which therefore is immunodominant. Although the location of protective epitopes in Slr remains unclear, the available data are compatible with the hypothesis that the LRR region is the key target for protective antibodies, focusing interest on this subdominant region for further studies of protective immunity elicited by immunization with Slr.

## Discussion

The development of a vaccine against *S. pyogenes* is defined as a priority by the WHO, reflecting the importance of this pathogen as the cause of human morbidity and mortality (World Health Organization, 2018). While a key problem in the development of an *S. pyogenes* vaccine is the potential for the vaccine to inadvertently cause rheumatic fever, which might be triggered by anti-streptococcal antibodies (Cunningham, 2019; Gray et al, 2017; Sheel et al, 2016), the identification and characterization of potential vaccine components remain essential for future prevention efforts. Our data show that Slr, the founding member of a family of streptococcal proteins, is a target for protective antibodies, implying that it is of interest as a vaccine candidate. Intriguingly, Slr is virtually absent from the surface of bacteria grown in broth but is turned on during infection, when it becomes a target for protective antibodies, as demonstrated in passive and active immunization experiments.

In our immunization experiments, mice were protected against lethal infection with *S. pyogenes* strains of several M types, indicating that Slr elicits broad protection. Passive immunization of mice with rabbit sera provided protection, demonstrating that anti-Slr antibodies alone are sufficient to confer protection. The observed protection was not complete but was similar to that reported in other studies of individual *S. pyogenes* vaccine candidates (Bensi et al, 2012; Fritzer et al, 2010; Henningham et al, 2012; Liu et al, 2007). Importantly, the incomplete protection we observed may be at least partly explained by the experimental protocol used, which involved the challenge of mice with bacteria that had been cultivated in broth and therefore lacked Slr at the time of infection. Our results contrast with previous studies in which Slr immunization did not confer protection (Fritzer et al, 2010) or conferred protection against one of three *S. pyogenes* strains (Bensi et al, 2012). These differences likely arise from differences in choice of challenge model (subcutaneous, intraperitoneal, intravenous) and adjuvant (which was Freund’s or alum in the published report) (Bensi et al, 2012; Fritzer et al, 2010). More fundamentally, our data defining AdcR as a regulator suggests that Slr expression in *S. pyogenes* is sensitive to environmental Zn. Therefore, differences in protection elicited by Slr immunization may also reflect different Zn levels and Slr expression by *S. pyogenes* in different tissue environments.

While it has remained unclear whether Slr is exposed at the bacterial surface under standard laboratory conditions, the flow cytometry analysis reported here shows that Slr is virtually absent from the surface of laboratory-cultivated *S. pyogenes* strains of various M types. Of note, this conclusion is supported by several biochemical studies, which did not detect Slr among the many surface proteins identified on *S. pyogenes* grown in broth (Bensi et al, 2012; Cole et al, 2005; Rodr**í**guez-Ortega et al, 2006; Severin et al, 2007). Despite the absence of Slr from the surface of laboratory-cultivated *S. pyogenes* strains, we found that it is a target for protective antibodies in infected mice, indicating that Slr is upregulated in vivo. Support for upregulation in vivo is provided by the demonstration that antibodies against Slr were elicited in *S. pyogenes*-infected macaques (Virtaneva et al, 2003) and by our analysis of serum samples from infected humans and mice, indicating that Slr elicits an antibody response comparable to that elicited by M protein. In addition, transcription studies show that transcription of the *slr* gene occurs at very low levels during growth in broth but is upregulated in vivo (Brenot et al, 2007; Hirose et al, 2019; Kachroo et al, 2020; Maamary et al, 2012b; Makthal et al, 2020; Sanson et al, 2015). For instance, *slr* was found to be among the most highly expressed genes in *S. pyogenes* clinical isolates recovered from patients with either pharyngeal or invasive disease (Sumby et al, 2006). Furthermore, RNA-seq experiments have identified *slr* as being significantly upregulated during skin infection in a mouse model (Wilkening et al, 2023). While transcription data must be interpreted with caution, since the presence of a transcript in vivo does not necessarily imply that the protein is translated (Freiberg et al, 2016), all available data now support the notion that the Slr protein is virtually absent from laboratory-cultivated bacteria but is upregulated during infection.

Though the extracellular signals that drive Slr upregulation in vivo are not fully understood, several transcription studies have indicated that expression of the *slr* gene is triggered by zinc deficiency (Brenot et al, 2007; Makthal et al, 2020; Sanson et al, 2015). In agreement with these reports, our data demonstrate that the Slr protein is virtually absent from the surface of wild-type bacteria cultivated in broth but is readily detected on the surface of bacteria lacking the repressor AdcR, which suppresses expression of zinc acquisition genes in environments with sufficient zinc (Sanson et al, 2015). A coherent picture is therefore emerging, establishing that zinc deficiency triggers the formation of the Slr protein in vivo. While earlier studies suggested that the presence of certain other surface structures limits exposure of Slr (Waldemarsson et al, 2006), those studies used a highly sensitive radioimmunoassay to study bacteria grown in broth, allowing detection of Slr even under conditions when it is expressed at background levels. Given the present insights about upregulation of Slr during zinc deficiency, the relevance of this earlier finding remains unclear.

An important aspect of our results is the support they provide for the classical, but sometimes overlooked, concept that pathogenic bacteria do not have the same properties in the laboratory as during an infection (Smith, 1958). Our data, therefore, focus interest on transcriptomic profiling by RNA-seq and related methods, as well as complementary library screening approaches such as in vivo expression technology (IVET), which may together allow the identification of vaccine targets upregulated in vivo (Chiang et al, 1999; Cummings and Relman, 2000; Lempke et al, 2023; Mahan et al, 1993a; Mahan et al, 1993b; Rediers et al, 2005; Westermann and Vogel, 2021). While IVET has so far been used to a relatively limited extent, powerful high-resolution transcriptomic and proteomic approaches that have the capability to provide single-cell or spatial data have recently arisen, which may enable novel screening in streptococci to identify additional candidates for future vaccine development.

Since Slr elicited a strong antibody response during infection, the question arises whether this anti-Slr response protects against new *S. pyogenes* infections. This is apparently not the case, since *S. pyogenes* typically causes repeated infections in humans, especially in children, implying that anti-Slr antibodies elicited during one infection do not efficiently protect against new infections. On the other hand, our immunization studies indicate that *S. pyogenes* infection can be prevented by antibodies to Slr. To explain this apparent paradox, we hypothesized that most antibodies elicited by Slr during an infection are directed against a region that is immunodominant but a poor target for protective antibodies, as previously described for the antibody response to several other microbial surface proteins (Lindahl, 2020; Stalhammar-Carlemalm et al, 2007). For Slr, the antibody response elicited during infection was indeed found to be directed almost entirely against one region, the HT region, although the LRR region probably is located distally from the bacterial surface and therefore may be most accessible to the immune system. Thus, the HT region is immunodominant, while the LRR region is subdominant. Independent support for the immunodominance of the HT region comes from a study in which human patient sera were used to screen *S. pyogenes* genomic libraries (Fritzer et al, 2010). Among 62 putative antibody targets identified in Slr, all were in the HT region, in agreement with the notion that this region is immunodominant. Importantly, immunodominance may represent a mechanism of immune evasion, allowing a pathogen to evade protective antibodies directed against a region that is subdominant (Lindahl, 2020). For Slr, the immunodominance of the HT region suggests that the subdominant LRR region might be a major target for protective antibodies, a hypothesis that has interesting implications for vaccine development.

Based on the arguments presented above, we propose a model, in which the antibody response to Slr during natural infection is directed towards the highly conserved HT region, allowing the subdominant LRR to evade antibodies and maintain function. This model is consistent with data on M proteins, for which the N-terminal hypervariable region (HVR) is subdominant but is a target for protective antibodies (Lannergård et al, 2011; Stalhammar-Carlemalm et al, 2007). We note that the specific protective efficacy of isolated LRR versus HT fragments needs to be formally analyzed in immunization and challenge studies. Regarding subdominance and protective immunity, it is of interest that Slr is the founding member of a family of streptococcal surface proteins with HT and LRR regions (Shao et al, 2013), implying that further studies of Slr may provide insights of relevance to the development of vaccines against other pathogens, including *Streptococcus agalactiae* and *Streptococcus dysgalactiae*. Since some of the Slr-like proteins cross-react (Waldemarsson et al, 2006), it is conceivable that antibodies to one Slr-like protein may protect against more than one pathogen, providing an additional impetus for studies of these proteins as vaccine candidates.

In summary, our study identifies Slr as a conserved, in vivo-induced antigen that is not expressed during standard laboratory growth but becomes a viable target for protective antibodies within the zinc-restricted environment of the host. Our characterization of the AdcR-mediated regulatory switch, and our demonstration that the antibody response is focused on the immunodominant HT region, allow us to mechanistically explain why this protective target has been largely overlooked in published screenings. Together, our findings support a model in which *S. pyogenes* utilizes both conditional expression and immune diversion to evade host defenses, and they highlight the interest of studying subdominance and in vivo expression for the rational design of novel vaccines.

## Methods

**Table:** Reagents and tools table

Reagent/resource	Reference or source	Identifier or catalog number
**Experimental models**		
*Mus musculus* C3H/HeN	Animal facility, Medical Faculty, Lund University	
*Oryctolagus cuniculus*	Local breeder Christer Månsson, Lund	
*S. pyogenes* strains	Table EV1	Table EV1
*E. coli* DH5α	Thermo Scientific	18258012
*E. coli* KJ622	Perez et al, 2000	
*E. coli* BL21	New England Biolabs	C2527H
*Oryctolagus cuniculus* sera:		
pre-immune	This study	
anti-Slr	This study	
anti-M5	This study	
anti-M5-HVR	This study	
anti-M1-HVR	This study	
anti-M3-HVR	This study	
anti-M22-HVR	This study	
*Mus musculus* sera:		
M1 infection serum	This study	
M3 infection serum	This study	
M5 infection serum	This study	
*Homo sapiens* sera:		
Patient 1 acute serum	This study	
Patient 1 convalescent serum	This study	
Patient 2 acute serum	This study	
patient 2 convalescent serum	This study	
**Recombinant DNA**		
pJRS233	Perez**-**Casal et al, 1993	
pDL278	LeBlanc et al, 1992	
**Antibodies**		
Rabbit anti-human IgG	Dako	GA512
Rabbit anti-mouse IgG	Sigma	M7023
Goat anti-rabbit IgG-HRP	Invitrogen	A16096
Goat anti-rabbit IgG-FITC	Invitrogen	A16097
**Oligonucleotides and other sequence-based reagents**		
PCR primers	Table EV2	Table EV2
**Chemicals, enzymes, and other reagents**		
Todd–Hewitt broth	Sigma	T1438-500G
Yeast extract	Oxoid	LP0021B
LB broth	Invitrogen	12780052
Ampicillin	SLS	MIC9024
Erythromycin	TCI	E0751
Kanamycin	Sigma	K4000-1G
Spectinomycin	Sigma	S742-5G
Phusion PCR Master Mix	Thermo Scientific	F-566
Gibson Assembly Master Mix	New England Biolabs	E2611L
Bam*HI*	New England Biolabs	R3136T
Sal*I*	New England Biolabs	R3138T
T4 ligase	New England Biolabs	M0202S
protein G	Sigma	539303
3,3’,5,5’-tetramethylbenzidine (TMB)	Invitrogen	00-4201-56
ECL substrate solution	Promega	W1015
CFA adjuvant	Sigma	AR001
IFA adjuvant	Sigma	AR002
CAF01 adjuvant	Christensen et al, 2009	
F(ab’)2 preparation kit	Pierce	44988
**Software**		
FlowJo	BD Biosciences	Version 10.8.0
GraphPad Prism	GraphPad Software	Version 10

### Ethics statement

All animal experiments, including immunization of mice and rabbits, and infections of mice, were performed with permission from the Animal Experimental Ethics Committee at Lund District Court. Experimental infections were performed in a level P2 biohazard laboratory within the animal facility of Department of Laboratory Medicine, Lund University, and were governed by relevant directives, laws and provisions: Council directive EG 86/609/EEC, the Swedish Animal Welfare Act (1988:534), the Swedish Animal Welfare Ordinance (1988:539), and provisions regarding the use of animals for scientific purposes: DFS 2004:15, DFS 2005:4, SJVFS 2001:91, SJVFS 1991:11. The serum samples from humans with invasive M1 infection were obtained with approval from the Research ethics committee at Karolinska Institutet and with written informed consent from the subjects or their legal guardians. Human blood was obtained from healthy donors, approved by the Regional Ethics Committee and Imperial College Healthcare NHS Trust Tissue Bank (Regional Ethics Committee approval no. 17/WA/0161, Imperial College Healthcare Tissue Bank Human Tissue Authority license no. 12275, and Imperial College Research Ethics Committee no. 19IC5166). All samples were collected after receiving signed informed consent from all participants. All experiments involving human subjects conform to the principles set out in the WMA Declaration of Helsinki and the Department of Health and Human Services Belmont Report.

### AlphaFold models

AlphaFold models of Slr (AF-Q99Z76-F1-model_v4) were acquired from the AlphaFold database in February 2025.

### Bacterial strains and culture conditions

The *S. pyogenes* strains used are listed in Table EV1. Cultures of *S. pyogenes* were cultivated without shaking in Todd–Hewitt broth supplemented with 1.5% yeast extract (TH-Y), in 5% CO_2_ at 37 °C. Cloning work in *Escherichia coli* employed strains DH5α and KJ622 (Perez et al, 2000) while strain BL21 was used for protein production. Strains of *E. coli* were grown with shaking at 37 °C in LB. When selection for antibiotic resistance was appropriate, *E. coli* strains were cultivated in LB supplemented with ampicillin (100 μg/ml), erythromycin (200 μg/ml), kanamycin (20 μg/ml) or spectinomycin (50 μg/ml), and *S. pyogenes* strains were cultivated in TH-Y with spectinomycin (50 μg/ml).

### Construction of *slr*-negative and *adcR*-negative mutants of strain M5 Manfredo

An *slr*-negative mutant was derived from strain M5 Manfredo, employing the *E. coli*–*Streptococcus* shuttle vector pJRS233 (Perez**-**Casal et al, 1993), which carries an erythromycin resistance gene. The methodology used was like that previously employed (Waldemarsson et al, 2006). Briefly, the 5’ and 3’ flanking regions of the *slr* gene were amplified with primers Δ*slr*F1, Δ*slr*R1, Δ*slr*F2, and Δ*slr*R2 (Table EV2) and used to construct a pJRS233 derivative in which most of the *slr* gene had been replaced with a kanamycin resistance cassette. This plasmid was transformed into strain M5 Manfredo, and the *Δslr* mutant was recovered after homologous recombination (Perez**-**Casal et al, 1993).

For the construction of an *adcR*-negative deletion mutant of strain M5 Manfredo, the shuttle vector pJRS233 (Perez**-**Casal et al, 1993) was employed to generate plasmid pJRS∆*adcR*. Briefly, 500 bp fragments flanking the *adcR* gene were amplified and spliced by overlap extension (SOE) PCR, using primers acdR_ko_F1+ acdR_ko_R1 and acdR_ko_F2+ acdR_ko_R2 (Table EV2). *Bam*HI and *Sal*I restriction sites were incorporated into the resulting PCR product to facilitate cloning into pJRS233. Plasmid pJRS*∆adcR* was transformed into strain M5 Manfredo by electroporation (Alves et al, 2023), and strain M5 Manfredo ∆*acdR* was recovered after homologous recombination PCR using primers outside of the cloned region to confirm deletion.

Strain M5 Manfredo ∆*acdR* was complemented using the vector pDL278 (LeBlanc et al, 1992) as described previously (Lynskey et al, 2013). Briefly, primers acdR_comp_F and acdR_comp_R (Table EV2) were used to amplify the *adcR* gene with 181 bp upstream sequence to include the native promoter. Bam*HI* sites were incorporated by PCR to facilitate cloning into pDL278, and the resulting plasmid was transformed into strain M5 Manfredo∆*acdR*.

### Recombinant proteins

Recombinant (r)Slr was derived from strain M5 Manfredo and purified as a GST-fusion, with removal of the tag before use, as described (Waldemarsson et al, 2006). This recombinant protein does not include the predicted signal peptide and lipobox of the protein, i.e., the N-terminal cysteine residue of the processed protein was also excluded, while the following 769 residues of Slr were included. rM1, rM3, rM5, and rM22 proteins, derived from the mature forms of the M proteins, have been described (Gustafsson et al, 2013; Lannergård et al, 2011).

Fragments of Slr derived from the HT and LRR regions were produced as GST-fusions after amplification of the relevant fragments from M5 Manfredo DNA, using primers Slr-F, HTR-R, LRR-F, and Slr-R (Table EV2). The rHT construct was derived from the N-terminal 397 aa of the processed form of Slr (but without the cysteine residue), while the rLRR construct corresponded to the C-terminal 372 aa. For binding and inhibition tests, these rSlr fragments were used uncleaved, i.e., with the GST tag, using free GST as a control.

### Antisera

Rabbit antisera to the streptococcal Slr and M5 proteins (both derived from strain M5 Manfredo) were raised by subcutaneous immunization with 100 μg protein in CFA, with two 50 μg boosters in IFA. Rabbit antisera to the hypervariable region (HVR) of the M1, M5, and M22 proteins have been described (Berggard et al, 2001; Lannergård et al, 2011; Lannergard et al, 2015), while antiserum to the M3-HVR was available in our collections (J.L. and G.L., unpublished). Antisera from mice infected with strains of different M types were recovered from C3H/HeN mice infected intraperitoneally 4 weeks earlier with a sublethal dose (~ 10^6^ colony-forming units (CFU)) of the *S. pyogenes* M1 strain SF370, the M3 strain 003Sm, or the M5 strain Manfredo. The paired human sera from patients with invasive *S. pyogenes* M1 infection have been described (Darenberg et al, 2003; Lannergård et al, 2011). The acute serum was recovered on day 1, and the convalescent serum on day 28 after inclusion in the study.

### Binding and inhibition assays with antisera and immobilized proteins

IgG binding assays with bacterial proteins using human and mouse sera were performed essentially as described (Lannergård et al, 2011). For analysis of the antibody response to Slr and M protein in humans infected with M1 strains and in mice infected with M1, M3, and M5 strains, microtiter wells were coated overnight at 4 °C with pure streptococcal protein, using a concentration determined in preliminary experiments to give optimal results. After incubation with the human and mouse serum samples, diluted as indicated, bound antibodies were detected by incubation with 1:2000 dilution of secondary antibodies (rabbit anti-human IgG or rabbit anti-mouse IgG; DakoCytomation, Denmark) and bound rabbit antibodies were subsequently detected by incubation with radiolabelled protein G. Background Fc-mediated binding (Mills and Ghosh, 2021) was negligible for these pure M proteins, as demonstrated in tests with normal human serum and normal mouse serum.

In inhibition assays, the ability of different proteins to block the binding of human or mouse anti-Slr to immobilized Slr was tested. Samples of the suitably diluted antiserum were mixed with increasing amounts of the inhibiting protein and incubated for 30 min at room temperature in a volume of 100 μl. The remaining binding activity was then analyzed as described for binding assays.

Binding assays with rabbit antisera to Slr and M5 analyzed the ability of these antibodies to bind Slr, M5, or Slr fragments. Microtiter wells were coated overnight at 4 °C with 10 μg/ml protein diluted in PBS. After incubation with the relevant antiserum, or control pre-immune serum, all diluted as indicated, the plates were washed and subsequently incubated with 0.2 µg/ml secondary antibody (goat anti-rabbit IgG-HRP, Invitrogen). Bound rabbit antibodies were subsequently detected using 3,3’,5,5’-tetramethylbenzidine (TMB) substrate. Absorbance was read at an optical density (OD) of 450 nm (OD_450_), and background absorbance at OD_650_ was subtracted before plotting the data.

### Passive and active immunization of mice and subsequent infection

In passive immunizations, C3H/HeN mice received intraperitoneal (i.p.) injections of rabbit anti-Slr (100 μl; diluted to 0.5 ml in PBS) 4 h before and just before the infection. Controls received i.p. injection of pre-immune rabbit serum. For evaluation of protection against lethal infection, the mice were infected i.p. with an ~LD_90_ dose (~ 5 × 10^7^ CFU) of exponentially growing *S. pyogenes* bacteria of the strain indicated. For evaluation of protection against growth in organs, passively immunized mice or control mice were challenged i.p. with a sublethal dose (~ 10^6^ CFU) of strain M5 Manfredo. The mice were sacrificed after 20 h, when the spleens were homogenized and analyzed for the presence of bacteria. In active immunizations, C3H/HeN mice were immunized subcutaneously with pure Slr, using 20 μg for the first immunization and 10 μg for a booster at 4 weeks. For each immunization, the antigen was delivered in a volume of 150 μl, of which 75 μl was protein in PBS and 75 μl was the liposome-based adjuvant CAF01, kindly supplied by Dr D Christensen (Abraham et al, 2019; Christensen et al, 2009). Controls received PBS and adjuvant. Two weeks after the booster, the mice were infected with an ~LD_90_ dose of exponentially growing M1 or M5 bacteria. For all infected mice, survival was recorded during a 4-day period. Animals were euthanized when appropriate, according to pre-determined criteria.

### Generation of F(ab’)2 fragments

For the generation of F(ab’)2 fragments from rabbit antibodies against Slr or M proteins, the immunoglobulin fraction was first purified from the corresponding antisera, using protein G affinity chromatography. The F(ab’)2 fragments were then generated using a F(ab’)2 preparation kit (Pierce), and reactivity of the generated F(ab’)2 fragments was assessed as described above for uncleaved rabbit antibodies.

### Flow cytometry assays

Surface exposure of Slr and M protein on *S. pyogenes* was analyzed by flow cytometry. Overnight cultures of bacteria were diluted 1:20 in TH-Y and grown to mid-exponential phase. The bacteria were harvested by centrifugation, washed twice in PBS with 0.5% bovine serum albumin (BSA), and the absorbance value. at OD_600_ of the suspension was adjusted to 0.4. Bacteria were mixed with antiserum diluted 1:300 and incubated on ice for 1 h. Where indicated, F(ab’)2 fragments, which had been generated as described, were used at 6 µg/ml. The bacteria were subsequently washed twice in PBS with 0.5% BSA. Pellets were resuspended in 1.5 µg/ml FITC-conjugated goat anti-rabbit IgG (Invitrogen). After 30 min, samples were washed twice and resuspended in PBS with 0.5% BSA. The samples were analyzed by flow cytometry on BD FACSCalibur.

### Whole blood killing assay

Bacterial strains were grown to mid-logarithmic growth phase (absorbance at OD_600_ 0.15 to 0.30), washed, resuspended, and diluted 1:5000 in PBS. 15 µl of bacterial suspension and 50 µl of serum were mixed and incubated at 37 °C for 30 min. Whole blood was drawn from healthy human donors with sodium heparin as an anticoagulant, 235 µl were added to the opsonized bacterial suspension, and incubated at 37 °C. After 3 h, serial dilutions were performed and plated out for CFU enumeration.

### Amino acid sequence of Slr

The amino acid sequence of Slr in different strains of *S. pyogenes* was derived from the nucleotide sequence, which was determined by standard methods. In strain M5 Manfredo, the reference strain used here, Slr has the same length as in strain MGAS5005, the strain used to first study Slr (Reid et al, 2003), and the sequences of the two proteins are 98.6% identical, with differences at 11 of the 792 residues in the unprocessed proteins.

### SDS-PAGE and western blot

SDS-PAGE and Western blot analysis with pure proteins were performed essentially as described (Lannergård et al, 2011; Waldemarsson et al, 2006). Extracts of *S. pyogenes* strains used for the detection of Slr were prepared from equivalent amounts of bacteria and analyzed as described (Waldemarsson et al, 2006). Briefly, bacterial samples were boiled in SDS-PAGE sample buffer, and after centrifugation, the supernatants were analyzed by Western blot, with rabbit anti-Slr serum diluted 1:3000 as the probe. Bound rabbit antibodies were detected by incubation of the blotting membrane with 3.3 µg/ml HRP-conjugated goat anti-rabbit (Invitrogen) followed by ECL substrate solution (Promega).

### Other methods

The data on expression of *slr* and *emm*1 were assessed from data available on the Gene Expression Omnibus (GEO–NCBI) under accession number GSE35568 using GEO2R (NCBI). The data were submitted and published by Maamary et al, 2012a (Maamary et al, 2012b). Multiple sequence alignment of Slr was performed using the PanX software dataset and package (Ding et al, 2018), and visualized using NCBI MSA Viewer 1.25.3.

### Statistics and reproducibility

Results from binding assays are presented as mean values with standard deviation (SD) from three independent determinations. In studies of passive and active immunization of mice, the results were displayed as Kaplan–Meier survival curves and analyzed by the log-rank test. In the analysis of protection against bacterial growth in organs, the horizontal lines within the boxes represent the median. The boxes represent the interquartile ranges, IQR, and the t-bars the lowest normal datum still within 1.5 IQR of the lower quartile, and the highest normal datum still within 1.5 IQR of the upper quartile. To compare CFU between groups, the Mann–Whitney *U* test was used. Except for animal infections, which, for ethical reasons, were in most cases performed only once (but together yielded unequivocal data), all experiments were performed at least twice, with similar results.

## Supplementary information

Table EV1

Table EV2

Table EV3

Peer Review File

Source data Fig. 1

Source data Fig. 2

Source data Fig. 3

Source data Fig. 4

Source data Fig. 5

Expanded View Figures

## Data Availability

All study data are included in the article and/or supporting information. The source data of this paper are collected in the following database record: biostudies:S-SCDT-10_1038-S44321-026-00470-0.

## References

[CR1] Abraham S, Juel HB, Bang P, Cheeseman HM, Dohn RB, Cole T, Kristiansen MP, Korsholm KS, Lewis D, Olsen AW et al (2019) Safety and immunogenicity of the chlamydia vaccine candidate CTH522 adjuvanted with CAF01 liposomes or aluminium hydroxide: a first-in-human, randomised, double-blind, placebo-controlled, phase 1 trial. Lancet Infect Dis 19:1091–1100 31416692 10.1016/S1473-3099(19)30279-8

[CR2] Adamou JE, Heinrichs JH, Erwin AL, Walsh W, Gayle T, Dormitzer M, Dagan R, Brewah YA, Barren P, Lathigra R et al (2001) Identification and characterization of a novel family of pneumococcal proteins that are protective against sepsis. Infect Immun 69:949–958 11159990 10.1128/IAI.69.2.949-958.2001 PMC97974

[CR4] Alves J, Rand JD, Johnston ABE, Bowen C, Lynskey NN (2023) Methylome-dependent transformation of emm1 group A streptococci. mBio 14:e0079823 37427929 10.1128/mbio.00798-23 PMC10470502

[CR6] Barnett TC, Bowen AC, Carapetis JR (2018) The fall and rise of Group A Streptococcus diseases. Epidemiol Infect 147:e4 30109840 10.1017/S0950268818002285 PMC6518539

[CR8] Bella J, Hindle KL, McEwan PA, Lovell SC (2008) The leucine-rich repeat structure. Cell Mol Life Sci 65:2307–2333 18408889 10.1007/s00018-008-8019-0 PMC11131621

[CR9] Bensi G, Mora M, Tuscano G, Biagini M, Chiarot E, Bombaci M, Capo S, Falugi F, Manetti AG, Donato P et al (2012) Multi high-throughput approach for highly selective identification of vaccine candidates: the Group A Streptococcus case. Mol Cell Proteom 11:M111.015693 10.1074/mcp.M111.015693 PMC3433891 22286755

[CR11] Berggard K, Johnsson E, Morfeldt E, Persson J, Stalhammar-Carlemalm M, Lindahl G (2001) Binding of human C4BP to the hypervariable region of M protein: a molecular mechanism of phagocytosis resistance in *Streptococcus pyogenes*. Mol Microbiol 42:539–551 11703674 10.1046/j.1365-2958.2001.02664.x

[CR12] Bersch B, Bougault C, Roux L, Favier A, Vernet T, Durmort C (2013) New insights into histidine triad proteins: solution structure of a *Streptococcus pneumoniae* PhtD domain and zinc transfer to AdcAII. PLoS ONE 8:e81168 24312273 10.1371/journal.pone.0081168 PMC3842936

[CR13] Bessen DE, Smeesters PR, Beall BW (2018) Molecular epidemiology, ecology, and evolution of group A Streptococci. Microbiol Spectr. 10.1128/microbiolspec.cpp3-0009-2018 10.1128/microbiolspec.cpp3-0009-2018 PMC11633622 30191802

[CR14] Bober M, Morgelin M, Olin AI, von Pawel-Rammingen U, Collin M (2011) The membrane bound LRR lipoprotein Slr, and the cell wall-anchored M1 protein from *Streptococcus pyogenes* both interact with type I collagen. PLoS ONE 6:e20345 21655249 10.1371/journal.pone.0020345 PMC3105044

[CR15] Bonazzi M, Lecuit M, Cossart P (2009) *Listeria monocytogenes* internalin and E-cadherin: from bench to bedside. Cold Spring Harb Perspect Biol 1:a003087 20066101 10.1101/cshperspect.a003087 PMC2773623

[CR16] Botos I, Segal DM, Davies DR (2011) The structural biology of Toll-like receptors. Structure 19:447–459 21481769 10.1016/j.str.2011.02.004 PMC3075535

[CR17] Brenot A, Weston BF, Caparon MG (2007) A PerR-regulated metal transporter (PmtA) is an interface between oxidative stress and metal homeostasis in *Streptococcus pyogenes*. Mol Microbiol 63:1185–1196 17238923 10.1111/j.1365-2958.2006.05577.x

[CR18] Brouwer S, Rivera-Hernandez T, Curren BF, Harbison-Price N, De Oliveira DMP, Jespersen MG, Davies MR, Walker MJ (2023) Pathogenesis, epidemiology and control of Group A Streptococcus infection. Nat Rev Microbiol 21:431–447 36894668 10.1038/s41579-023-00865-7 PMC9998027

[CR19] Cannon JW, Jack S, Wu Y, Zhang J, Baker MG, Geelhoed E, Fraser J, Carapetis JR (2018) An economic case for a vaccine to prevent group A streptococcus skin infections. Vaccine 36:6968–6978 30340879 10.1016/j.vaccine.2018.10.001

[CR20] Carapetis JR, Steer AC, Mulholland EK, Weber M (2005) The global burden of group A streptococcal diseases. Lancet Infect Dis 5:685–694 16253886 10.1016/S1473-3099(05)70267-X

[CR21] Chiang SL, Mekalanos JJ, Holden DW (1999) In vivo genetic analysis of bacterial virulence. Annu Rev Microbiol 53:129–154 10547688 10.1146/annurev.micro.53.1.129

[CR22] Christensen D, Agger EM, Andreasen LV, Kirby D, Andersen P, Perrie Y (2009) Liposome-based cationic adjuvant formulations (CAF): past, present, and future. J Liposome Res 19:2–11 19515003 10.1080/08982100902726820

[CR23] Cole JN, Ramirez RD, Currie BJ, Cordwell SJ, Djordjevic SP, Walker MJ (2005) Surface analyses and immune reactivities of major cell wall-associated proteins of group A Streptococcus. Infect Immun 73:3137–3146 15845522 10.1128/IAI.73.5.3137-3146.2005 PMC1087385

[CR24] Cummings CA, Relman DA (2000) Using DNA microarrays to study host-microbe interactions. Emerg Infect Dis 6:513–525 10998383 10.3201/eid0605.000511 PMC2627958

[CR25] Cunningham MW (2019) Molecular mimicry, autoimmunity, and infection: the cross-reactive antigens of Group A Streptococci and their sequelae. Microbiol Spectr. 10.1128/microbiolspec.GPP3-0045-2018 10.1128/microbiolspec.gpp3-0045-2018 PMC6684244 31373269

[CR26] Darenberg J, Ihendyane N, Sjölin J, Aufwerber E, Haidl S, Follin P, Andersson J, Norrby-Teglund A (2003) Intravenous immunoglobulin G therapy in streptococcal toxic shock syndrome: a European randomized, double-blind, placebo-controlled trial. Clin Infect Dis 37:333–340 12884156 10.1086/376630

[CR27] Denny FW Jr., Perry WD, Wannamaker LW (1957) Type-specific streptococcal antibody. J Clin Invest 36:1092–1100 13449161 10.1172/JCI103504 PMC1072695

[CR28] Ding W, Baumdicker F, Neher RA (2018) panX: pan-genome analysis and exploration. Nucleic Acids Res 46:e5 29077859 10.1093/nar/gkx977 PMC5758898

[CR29] Ferretti JJ, McShan WM, Ajdic D, Savic DJ, Savic G, Lyon K, Primeaux C, Sezate S, Suvorov AN, Kenton S et al (2001) Complete genome sequence of an M1 strain of *Streptococcus pyogenes*. Proc Natl Acad Sci USA 98:4658–4663 11296296 10.1073/pnas.071559398 PMC31890

[CR30] Fischetti VA (1989) Streptococcal M protein: molecular design and biological behavior. Clin Microbiol Rev 2:285–314 2670192 10.1128/cmr.2.3.285 PMC358122

[CR31] Freiberg JA, Le Breton Y, Tran BQ, Scott AJ, Harro JM, Ernst RK, Goo YA, Mongodin EF, Goodlett DR, McIver KS et al (2016) Global analysis and comparison of the transcriptomes and proteomes of Group A Streptococcus biofilms. mSystems 1:e00149–16 27933318 10.1128/mSystems.00149-16 PMC5141267

[CR32] Fritzer A, Senn BM, Minh DB, Hanner M, Gelbmann D, Noiges B, Henics T, Schulze K, Guzman CA, Goodacre J et al (2010) Novel conserved group A streptococcal proteins identified by the antigenome technology as vaccine candidates for a non-M protein-based vaccine. Infect Immun 78:4051–4067 20624906 10.1128/IAI.00295-10 PMC2937439

[CR33] Good MF (2020) Streptococcus: an organism causing diseases beyond neglect. PLoS Negl Trop Dis 14:e0008095 32437344 10.1371/journal.pntd.0008095 PMC7241690

[CR35] Gray LA, D’Antoine HA, Tong SYC, McKinnon M, Bessarab D, Brown N, Reményi B, Steer A, Syn G, Blackwell JM et al (2017) Genome-wide analysis of genetic risk factors for rheumatic heart disease in aboriginal australians provides support for pathogenic molecular mimicry. J Infect Dis 216:1460–1470 29029143 10.1093/infdis/jix497

[CR37] Gustafsson MC, Lannergard J, Nilsson OR, Kristensen BM, Olsen JE, Harris CL, Ufret-Vincenty RL, Stalhammar-Carlemalm M, Lindahl G (2013) Factor H binds to the hypervariable region of many *Streptococcus pyogenes* M proteins but does not promote phagocytosis resistance or acute virulence. PLoS Pathog 9:e1003323 23637608 10.1371/journal.ppat.1003323 PMC3630203

[CR38] Harbison-Price N, Rivera-Hernandez T, Osowicki J, Davies MR, Steer AC, Walker MJ, Dale JB (2022) Current approaches to vaccine development of *Streptococcus pyogenes*. In: Ferretti JJ, Stevens DL, Fischetti VA (eds) *Streptococcus pyogenes*: Basic biology to clinical manifestations. Oklahoma City: University of Oklahoma Health Sciences Center. 837–917 36479760

[CR39] Henningham A, Chiarot E, Gillen CM, Cole JN, Rohde M, Fulde M, Ramachandran V, Cork AJ, Hartas J, Magor G et al (2012) Conserved anchorless surface proteins as group A Streptococcal vaccine candidates. J Mol Med 90:1197–1207 22527883 10.1007/s00109-012-0897-9

[CR40] Henningham A, Gillen CM, Walker MJ (2013) Group A streptococcal vaccine candidates: potential for the development of a human vaccine. Curr Top Microbiol Immunol 368:207–242 23250780 10.1007/82_2012_284

[CR41] Hirose Y, Yamaguchi M, Okuzaki D, Motooka D, Hamamoto H, Hanada T, Sumitomo T, Nakata M, Kawabata S (2019) *Streptococcus pyogenes* transcriptome changes in the inflammatory environment of necrotizing fasciitis. Appl Environ Microbiol 85:e01428–19 31471300 10.1128/AEM.01428-19 PMC6803311

[CR43] Kachroo P, Eraso JM, Olsen RJ, Zhu L, Kubiak SL, Pruitt L, Yerramilli P, Cantu CC, Ojeda Saavedra M, Pensar J et al (2020) New pathogenesis mechanisms and translational leads identified by multidimensional analysis of necrotizing myositis in primates. mBio 11:e03363–19 32071274 10.1128/mBio.03363-19 PMC7029145

[CR44] Keeley AJ, Camara FE, Armitage EP, de Crombrugghe G, Sillah J, Fofana ML, Rollinson V, Senghore E, Jammeh M, Whitcombe AL et al (2025) Early-life serological profiles and the development of natural protective humoral immunity to *Streptococcus pyogenes* in a high-burden setting. Nat Med 31:3360–3371 40781379 10.1038/s41591-025-03868-4 PMC12532705

[CR45] Lannergård J, Gustafsson MC, Waldemarsson J, Norrby-Teglund A, Stålhammar-Carlemalm M, Lindahl G (2011) The hypervariable region of *Streptococcus pyogenes* M protein escapes antibody attack by antigenic variation and weak immunogenicity. Cell Host Microbe 10:147–157 21843871 10.1016/j.chom.2011.06.011

[CR46] Lannergard J, Kristensen BM, Gustafsson MC, Persson JJ, Norrby-Teglund A, Stalhammar-Carlemalm M, Lindahl G (2015) Sequence variability is correlated with weak immunogenicity in *Streptococcus pyogenes* M protein. Microbiologyopen 4:774–789 26175306 10.1002/mbo3.278 PMC4618610

[CR47] LeBlanc DJ, Lee LN, Abu-Al-Jaibat A (1992) Molecular, genetic, and functional analysis of the basic replicon of pVA380-1, a plasmid of oral streptococcal origin. Plasmid 28:130–145 1409970 10.1016/0147-619x(92)90044-b

[CR48] Lempke S, May D, Ewald SE (2023) Microbial pathogenesis in the era of spatial omics. Infect Immun 91:e0044222 37255461 10.1128/iai.00442-22 PMC10353406

[CR49] Lindahl G (2020) Subdominance in antibody responses: implications for vaccine development. Microbiol Mol Biol Rev 85:e00078–20 33239435 10.1128/MMBR.00078-20 PMC7709521

[CR50] Liu M, Zhu H, Zhang J, Lei B (2007) Active and passive immunizations with the streptococcal esterase Sse protect mice against subcutaneous infection with group A Streptococci. Infect Immun 75:3651–3657 17502395 10.1128/IAI.00038-07 PMC1932925

[CR51] Luo Z, Pederick VG, Paton JC, McDevitt CA, Kobe B (2018) Structural characterisation of the HT3 motif of the polyhistidine triad protein D from *Streptococcus pneumoniae*. FEBS Lett 592:2341–2350 29856892 10.1002/1873-3468.13122

[CR52] Lynskey NN, Goulding D, Gierula M, Turner CE, Dougan G, Edwards RJ, Sriskandan S (2013) RocA truncation underpins hyper-encapsulation, carriage longevity and transmissibility of serotype M18 group A Streptococci. PLoS Pathog 9:e1003842 24367267 10.1371/journal.ppat.1003842 PMC3868526

[CR53] Maamary PG, Barnett TC, Walker MJ (2012a) Transcriptomic comparison of serotype M1 *S. pyogenes*. GSE35568 (https://www.ncbi.nlm.nih.gov/geo/query/acc.cgi?acc=GSE35568) [DATASET]

[CR54] Maamary PG, Ben Zakour NL, Cole JN, Hollands A, Aziz RK, Barnett TC, Cork AJ, Henningham A, Sanderson-Smith M, McArthur JD et al (2012b) Tracing the evolutionary history of the pandemic group A Streptococcal M1T1 clone. FASEB J 26:4675–4684 22878963 10.1096/fj.12-212142 PMC3475248

[CR55] Mahan MJ, Slauch JM, Hanna PC, Camilli A, Tobias JW, Waldor MK, Mekalanos JJ (1993a) Selection for bacterial genes that are specifically induced in host tissues: the hunt for virulence factors. Infect Agents Dis 2:263–268 8173806

[CR56] Mahan MJ, Slauch JM, Mekalanos JJ (1993b) Selection of bacterial virulence genes that are specifically induced in host tissues. Science 259:686–688 8430319 10.1126/science.8430319

[CR57] Makthal N, Do H, Wendel BM, Olsen RJ, Helmann JD, Musser JM, Kumaraswami M (2020) Group A Streptococcus AdcR regulon participates in bacterial defense against host-mediated zinc sequestration and contributes to virulence. Infect Immun 88:e00097–20 32393509 10.1128/IAI.00097-20 PMC7375770

[CR58] Makthal N, Kumaraswami M (2017) Zinc’ing it out: zinc homeostasis mechanisms and their impact on the pathogenesis of human pathogen group A Streptococcus. Metallomics 9:1693–1702 29043347 10.1039/c7mt00240h PMC5730477

[CR59] Medina E, Goldmann O, Rohde M, Lengeling A, Chhatwal GS (2001) Genetic control of susceptibility to group A streptococcal infection in mice. J Infect Dis 184:846–852 11550125 10.1086/323292

[CR60] Mills JO, Ghosh P (2021) Nonimmune antibody interactions of Group A Streptococcus M and M-like proteins. PLoS Pathog 17:e1009248 33630944 10.1371/journal.ppat.1009248 PMC7906336

[CR61] Mortensen R, Nissen TN, Fredslund S, Rosenkrands I, Christensen JP, Andersen P, Dietrich J (2016) Identifying protective *Streptococcus pyogenes* vaccine antigens recognized by both B and T cells in human adults and children. Sci Rep 6:22030 26911649 10.1038/srep22030 PMC4766568

[CR62] Panina EM, Mironov AA, Gelfand MS (2003) Comparative genomics of bacterial zinc regulons: enhanced ion transport, pathogenesis, and rearrangement of ribosomal proteins. Proc Natl Acad Sci USA 100:9912–9917 12904577 10.1073/pnas.1733691100 PMC187884

[CR63] Pastural E, McNeil SA, MacKinnon-Cameron D, Ye L, Langley JM, Stewart R, Martin LH, Hurley GJ, Salehi S, Penfound TA et al (2020) Safety and immunogenicity of a 30-valent M protein-based group A streptococcal vaccine in healthy adult volunteers: a randomized, controlled phase I study. Vaccine 38:1384–1392 31843270 10.1016/j.vaccine.2019.12.005

[CR64] Perez AR, Abanes-De Mello A, Pogliano K (2000) SpoIIB localizes to active sites of septal biogenesis and spatially regulates septal thinning during engulfment in *Bacillus subtilis*. J Bacteriol 182:1096–1108 10648537 10.1128/jb.182.4.1096-1108.2000 PMC94387

[CR65] Perez-Casal J, Price JA, Maguin E, Scott JR (1993) An M protein with a single C repeat prevents phagocytosis of *Streptococcus pyogenes*: use of a temperature-sensitive shuttle vector to deliver homologous sequences to the chromosome of *S. pyogenes*. Mol Microbiol 8:809–819 8355608 10.1111/j.1365-2958.1993.tb01628.x

[CR66] Pfoh E, Wessels MR, Goldmann D, Lee GM (2008) Burden and economic cost of group A Streptococcal pharyngitis. Pediatrics 121:229–234 18245412 10.1542/peds.2007-0484

[CR67] Plumptre CD, Ogunniyi AD, Paton JC (2012) Polyhistidine triad proteins of pathogenic streptococci. Trends Microbiol 20:485–493 22819099 10.1016/j.tim.2012.06.004

[CR68] Rediers H, Rainey PB, Vanderleyden J, De Mot R (2005) Unraveling the secret lives of bacteria: use of in vivo expression technology and differential fluorescence induction promoter traps as tools for exploring niche-specific gene expression. Microbiol Mol Biol Rev 69:217–261 15944455 10.1128/MMBR.69.2.217-261.2005 PMC1197422

[CR69] Reglinski M, Lynskey NN, Choi YJ, Edwards RJ, Sriskandan S (2016) Development of a multicomponent vaccine for *Streptococcus pyogenes* based on the antigenic targets of IVIG. J Infect 72:450–459 26880087 10.1016/j.jinf.2016.02.002 PMC4796040

[CR70] Reid SD, Green NM, Buss JK, Lei B, Musser JM (2001) Multilocus analysis of extracellular putative virulence proteins made by group A Streptococcus: population genetics, human serologic response, and gene transcription. Proc Natl Acad Sci USA 98:7552–7557 11416223 10.1073/pnas.121188598 PMC34706

[CR71] Reid SD, Montgomery AG, Voyich JM, DeLeo FR, Lei B, Ireland RM, Green NM, Liu M, Lukomski S, Musser JM (2003) Characterization of an extracellular virulence factor made by group A Streptococcus with homology to the *Listeria monocytogenes* internalin family of proteins. Infect Immun 71:7043–7052 14638794 10.1128/IAI.71.12.7043-7052.2003 PMC308899

[CR72] Riboldi-Tunnicliffe A, Isaacs NW, Mitchell TJ (2005) 1.2 Angstroms crystal structure of the *S. pneumoniae* PhtA histidine triad domain a novel zinc binding fold. FEBS Lett 579:5353–5360 16194532 10.1016/j.febslet.2005.08.066

[CR73] Rivera-Hernandez T, Pandey M, Henningham A, Cole J, Choudhury B, Cork AJ, Gillen CM, Ghaffar KA, West NP, Silvestri G et al (2016) Differing efficacies of lead group A Streptococcal vaccine candidates and full-length M protein in cutaneous and invasive disease models. mBio 7:e00618–16 27302756 10.1128/mBio.00618-16 PMC4916377

[CR74] Rivera-Hernandez T, Rhyme MS, Cork AJ, Jones S, Segui-Perez C, Brunner L, Richter J, Petrovsky N, Lawrenz M, Goldblatt D et al (2020) Vaccine-induced Th1-type response protects against invasive group A Streptococcus infection in the absence of opsonizing antibodies. mBio 11:e00122–20 32156809 10.1128/mBio.00122-20 PMC7064752

[CR75] Rodríguez-Ortega MJ, Norais N, Bensi G, Liberatori S, Capo S, Mora M, Scarselli M, Doro F, Ferrari G, Garaguso I et al (2006) Characterization and identification of vaccine candidate proteins through analysis of the group A Streptococcus surface proteome. Nat Biotechnol 24:191–197 16415855 10.1038/nbt1179

[CR76] Sabet C, Lecuit M, Cabanes D, Cossart P, Bierne H (2005) LPXTG protein InlJ, a newly identified internalin involved in *Listeria monocytogenes* virulence. Infect Immun 73:6912–6922 16177371 10.1128/IAI.73.10.6912-6922.2005 PMC1230919

[CR77] Sanduja P, Gupta M, Somani VK, Yadav V, Dua M, Hanski E, Sharma A, Bhatnagar R, Johri AK (2020) Cross-serotype protection against group A Streptococcal infections induced by immunization with SPy_2191. Nat Commun 11:3545 32669564 10.1038/s41467-020-17299-x PMC7363907

[CR78] Sanson M, Makthal N, Flores AR, Olsen RJ, Musser JM, Kumaraswami M (2015) Adhesin competence repressor (AdcR) from *Streptococcus pyogenes* controls adaptive responses to zinc limitation and contributes to virulence. Nucleic Acids Res 43:418–432 25510500 10.1093/nar/gku1304 PMC4288194

[CR79] Sati H, Carrara E, Savoldi A, Hansen P, Garlasco J, Campagnaro E, Boccia S, Castillo-Polo JA, Magrini E, Garcia-Vello P et al (2025) The WHO Bacterial Priority Pathogens List 2024: a prioritisation study to guide research, development, and public health strategies against antimicrobial resistance. Lancet Infect Dis 25:1033–1043 40245910 10.1016/S1473-3099(25)00118-5 PMC12367593

[CR80] Severin A, Nickbarg E, Wooters J, Quazi SA, Matsuka YV, Murphy E, Moutsatsos IK, Zagursky RJ, Olmsted SB (2007) Proteomic analysis and identification of *Streptococcus pyogenes* surface-associated proteins. J Bacteriol 189:1514–1522 17142387 10.1128/JB.01132-06 PMC1855729

[CR81] Shao ZQ, Zhang YM, Pan XZ, Wang B, Chen JQ (2013) Insight into the evolution of the histidine triad protein (HTP) family in Streptococcus. PLoS ONE 8:e60116 23527301 10.1371/journal.pone.0060116 PMC3603884

[CR82] Sheel M, Moreland NJ, Fraser JD, Carapetis J (2016) Development of Group A streptococcal vaccines: an unmet global health need. Expert Rev Vaccines 15:227–238 26559880 10.1586/14760584.2016.1116946

[CR83] Smith H (1958) The use of bacteria grown in vivo for studies on the basis of their pathogenicity. Annu Rev Microbiol 12:77–102 13595602 10.1146/annurev.mi.12.100158.000453

[CR84] Stalhammar-Carlemalm M, Waldemarsson J, Johnsson E, Areschoug T, Lindahl G (2007) Nonimmunodominant regions are effective as building blocks in a streptococcal fusion protein vaccine. Cell Host Microbe 2:427–434 18078694 10.1016/j.chom.2007.10.003

[CR85] Stenberg L, O’Toole P, Lindahl G (1992) Many group A streptococcal strains express two different immunoglobulin-binding proteins, encoded by closely linked genes: characterization of the proteins expressed by four strains of different M-type. Mol Microbiol 6:1185–1194 1588817 10.1111/j.1365-2958.1992.tb01557.x

[CR86] Sumby P, Whitney AR, Graviss EA, DeLeo FR, Musser JM (2006) Genome-wide analysis of group A streptococci reveals a mutation that modulates global phenotype and disease specificity. PLoS Pathog 2:e5 16446783 10.1371/journal.ppat.0020005 PMC1354197

[CR87] Sutcliffe IC, Harrington DJ (2002) Pattern searches for the identification of putative lipoprotein genes in Gram-positive bacterial genomes. Microbiology 148:2065–2077 12101295 10.1099/00221287-148-7-2065

[CR88] Turner AG, Ong CY, Walker MJ, Djoko KY, McEwan AG (2017) Transition metal homeostasis in *Streptococcus pyogenes* and *Streptococcus pneumoniae*. Adv Micro Physiol 70:123–191 10.1016/bs.ampbs.2017.01.002 28528647

[CR89] Virtaneva K, Graham MR, Porcella SF, Hoe NP, Su H, Graviss EA, Gardner TJ, Allison JE, Lemon WJ, Bailey JR et al (2003) Group A Streptococcus gene expression in humans and cynomolgus macaques with acute pharyngitis. Infect Immun 71:2199–2207 12654842 10.1128/IAI.71.4.2199-2207.2003 PMC152081

[CR90] Waldemarsson J, Areschoug T, Lindahl G, Johnsson E (2006) The streptococcal Blr and Slr proteins define a family of surface proteins with leucine-rich repeats: camouflaging by other surface structures. J Bacteriol 188:378–388 16385027 10.1128/JB.188.2.378-388.2006 PMC1347292

[CR91] Walkinshaw DR, Wright MEE, Mullin AE, Excler JL, Kim JH, Steer AC (2023) The *Streptococcus pyogenes* vaccine landscape. NPJ Vaccines 8:16 36788225 10.1038/s41541-023-00609-x PMC9925938

[CR92] Westermann AJ, Vogel J (2021) Cross-species RNA-seq for deciphering host-microbe interactions. Nat Rev Genet 22:361–378 33597744 10.1038/s41576-021-00326-y

[CR93] Whatmore AM, Kehoe MA (1994) Horizontal gene transfer in the evolution of group A streptococcal emm-like genes: gene mosaics and variation in Vir regulons. Mol Microbiol 11:363–374 8170398 10.1111/j.1365-2958.1994.tb00316.x

[CR94] Wilkening RV, Langouet-Astrie C, Severn MM, Federle MJ, Horswill AR (2023) Identifying genetic determinants of *Streptococcus pyogenes*-host interactions in a murine intact skin infection model. Cell Rep 42:113332 37889753 10.1016/j.celrep.2023.113332 PMC10841832

[CR95] World Health Organization (2018) Group A streptococcus vaccine development technology roadmap: priority activities for development, testing, licensure and global availability of group A streptococcus vaccines. World Health Organization, Geneva.

