# Supplementary material for: The Slr protein of Streptococcus pyogenes is selectively expressed in vivo and is a target for protective antibodies
Source: EMBO Mol Med. 2026 Jun 15;18(8):3086–108. doi: 10.1038/s44321-026-00470-0 (PMC13470328; doi:10.1038/s44321-026-00470-0)
Supplement: Supplementary file 1 [file 44321_2026_470_MOESM10_ESM.pdf]

Expanded View Figures

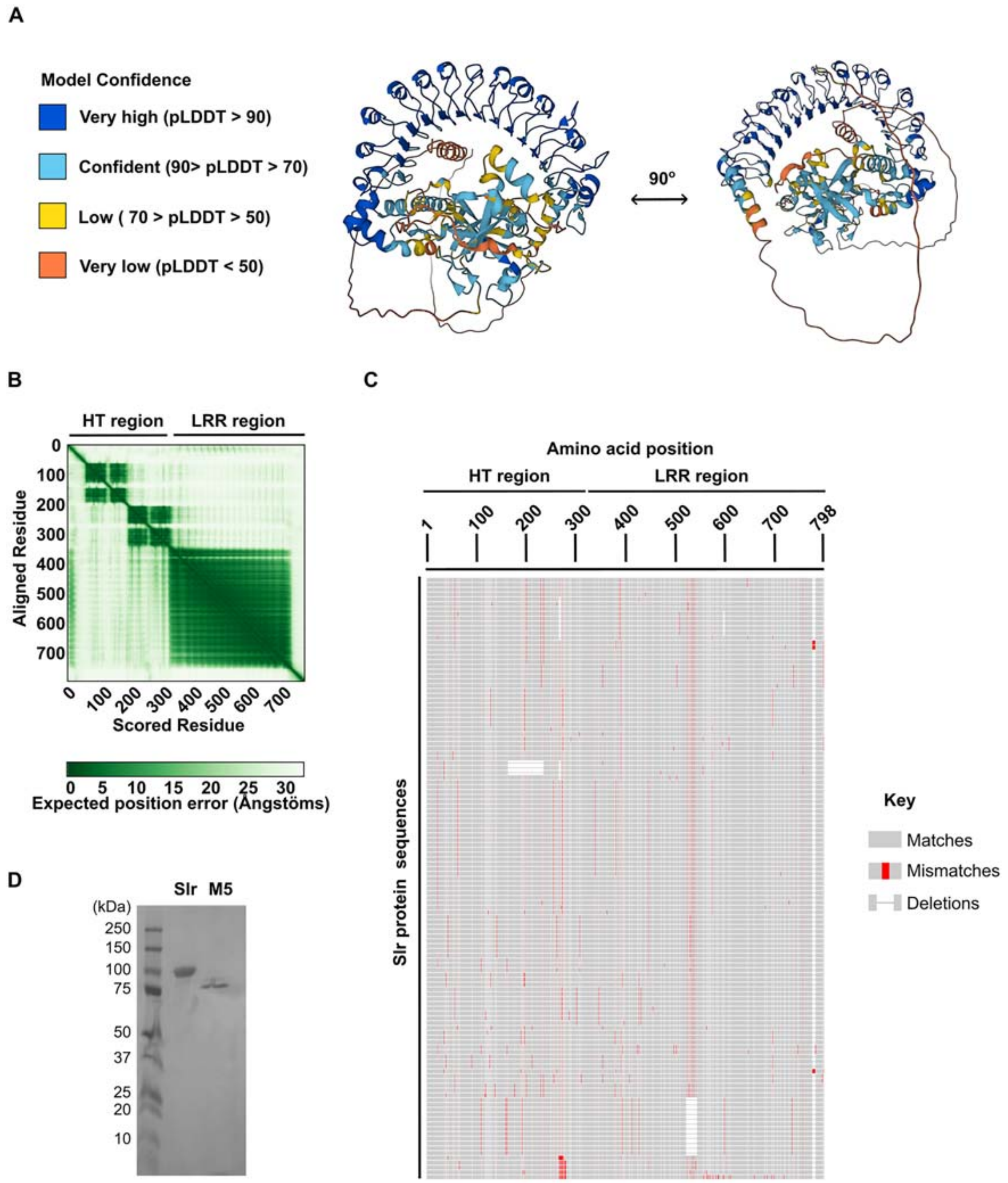

Figure EV1. Structure of the Slr protein and sequence conservation.

(**A**) AlphaFold prediction for Slr (Accession: AF-Q99Z76-F1), shown in two orientations. The prediction is colored by the model confidence band. (**B**) The expected positional error panel of the predicted Slr structure shows dark green patches for the four histidine triad (HT) domains and leucine repeat region (LRR), indicating that they are well-defined. Note that the green shading is weak between the four HT domains and between the HT and LRR regions, indicating that the inter-domain configuration is not well-defined. (**C**) Multiple sequence analysis of Slr from 125 *S. pyogenes* genomes on PanX software package, visualized using NCBI MSA Viewer 1.25.3. Each horizontal line represents one Slr protein sequence. Variation in sequence is represented by red coloring, with the shade of red indicative of frequency. (**D**) Expression and purification of recombinant Slr, compared with pure recombinant M5.

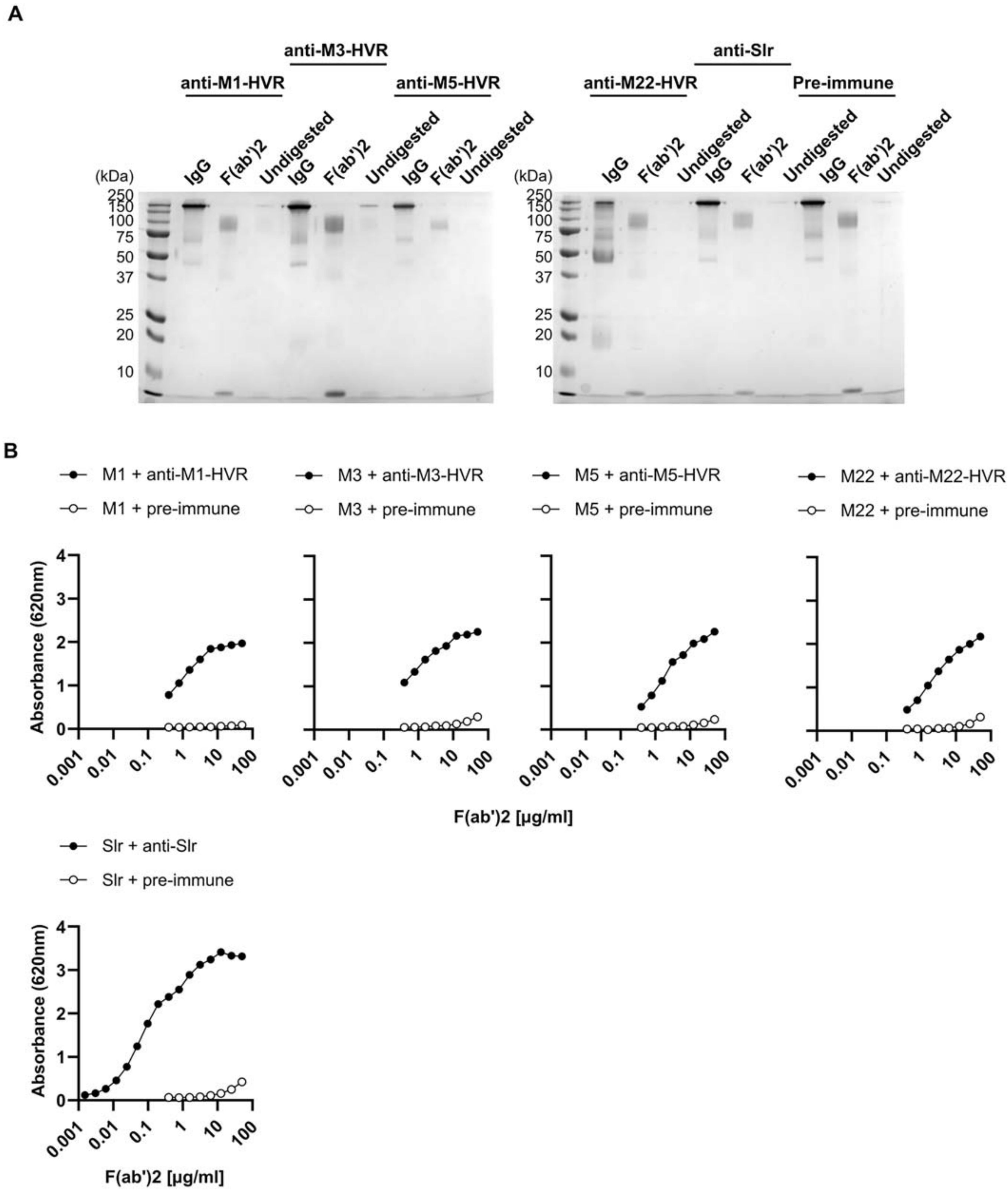
A
anti-M3-HVR
anti-M1-HVR
anti-M5-HVR
anti-Slr
anti-M22-HVR
Pre-immune
IgG
F(ab')2
Undigested
(kDa)
250
150
100
75
50
37
25
20
10
B
M1 + anti-M1-HVR
M1 + pre-immune
M3 + anti-M3-HVR
M3 + pre-immune
M5 + anti-M5-HVR
M5 + pre-immune
M22 + anti-M22-HVR
M22 + pre-immune
Absorbance (620nm)
0
1
2
3
4
0.001
0.01
0.1
1
10
100
F(ab')2 [µg/ml]
Slr + anti-Slr
Slr + pre-immune

 © The Author(s)

Figure EV2. Purification and reactivity of F(ab′)2 fragments derived from anti-M-HVR and anti-Slr.

(**A**) IgG was purified from rabbit antisera (raised against M1-HVR, M3-HVR, M5-HVR, M22-HVR, or against Slr) and from pre-immune rabbit serum, using a protein G column. F(ab′)2 fragments were prepared by digestion of purified IgG with pepsin and purified using protein A columns. Input IgG, F(ab′)2, and the unsuccessfully digested portion of the input IgG were separated by SDS-PAGE analysis. (**B**) To verify that the F(ab′)2 fragments generated from rabbit anti-M (HVR) and anti-Slr antibodies retained reactivity to the corresponding intact M proteins or Slr, they were analyzed, as indicated, for reactivity with the pure M protein or Slr immobilized in microtiter wells. F(ab′)2 fragments derived from pre-immune IgG were used as a control. F(ab′)2 interaction with immobilized proteins was detected by ELISA. Means ± SD from $n = 3$ independent experiments.

© The Author(s)

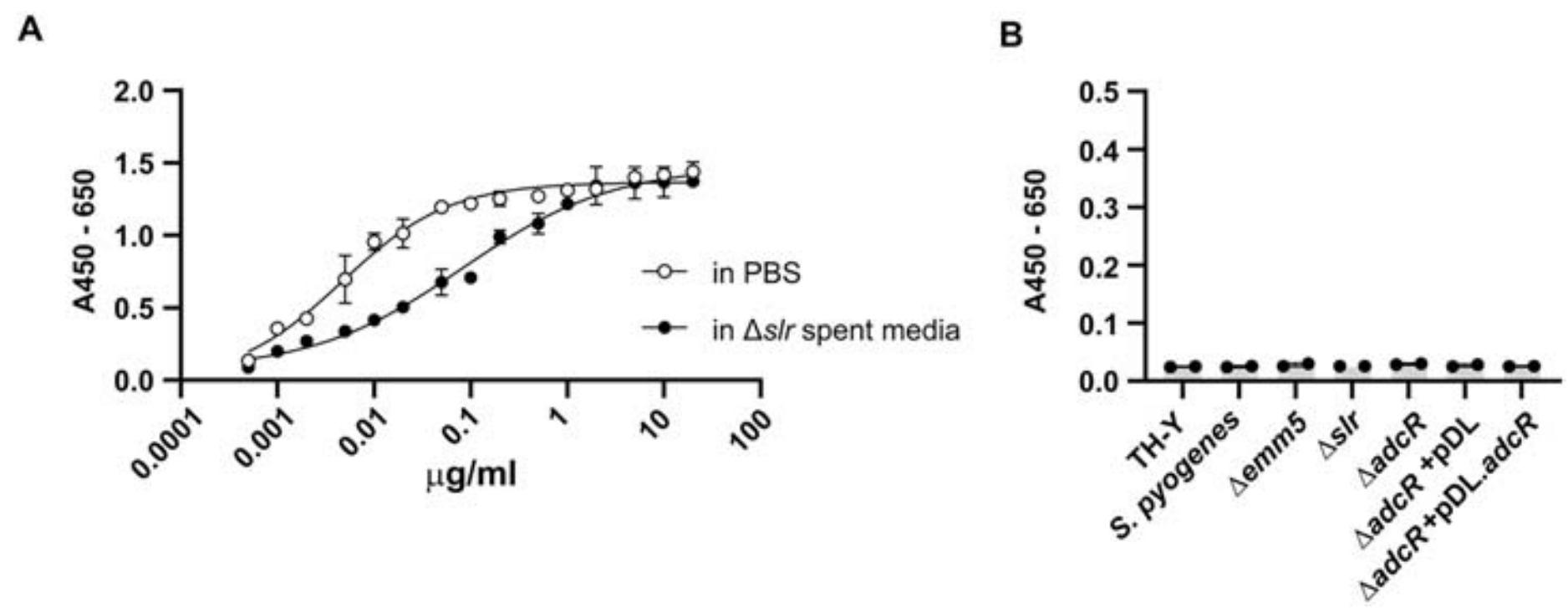

Figure EV3. Slr is not detected in *S. pyogenes* culture supernatants.

(**A**) ELISA used to detect Slr. Pure Slr was diluted in PBS or in spent TH-Y media (recovered from a culture of the *S. pyogenes* Δ*slr* strain), diluted to the concentrations shown, and coated onto microtiter plates. Reactivity with rabbit anti-Slr IgG was tested. Means ± SD from $n = 3$ technical replicates. (**B**) Detection of Slr in the supernatants of *S. pyogenes* by ELISA. Culture supernatants were coated onto microtiter plates and tested for reactivity with rabbit anti-Slr IgG. Two independent experiments are shown.

© The Author(s)

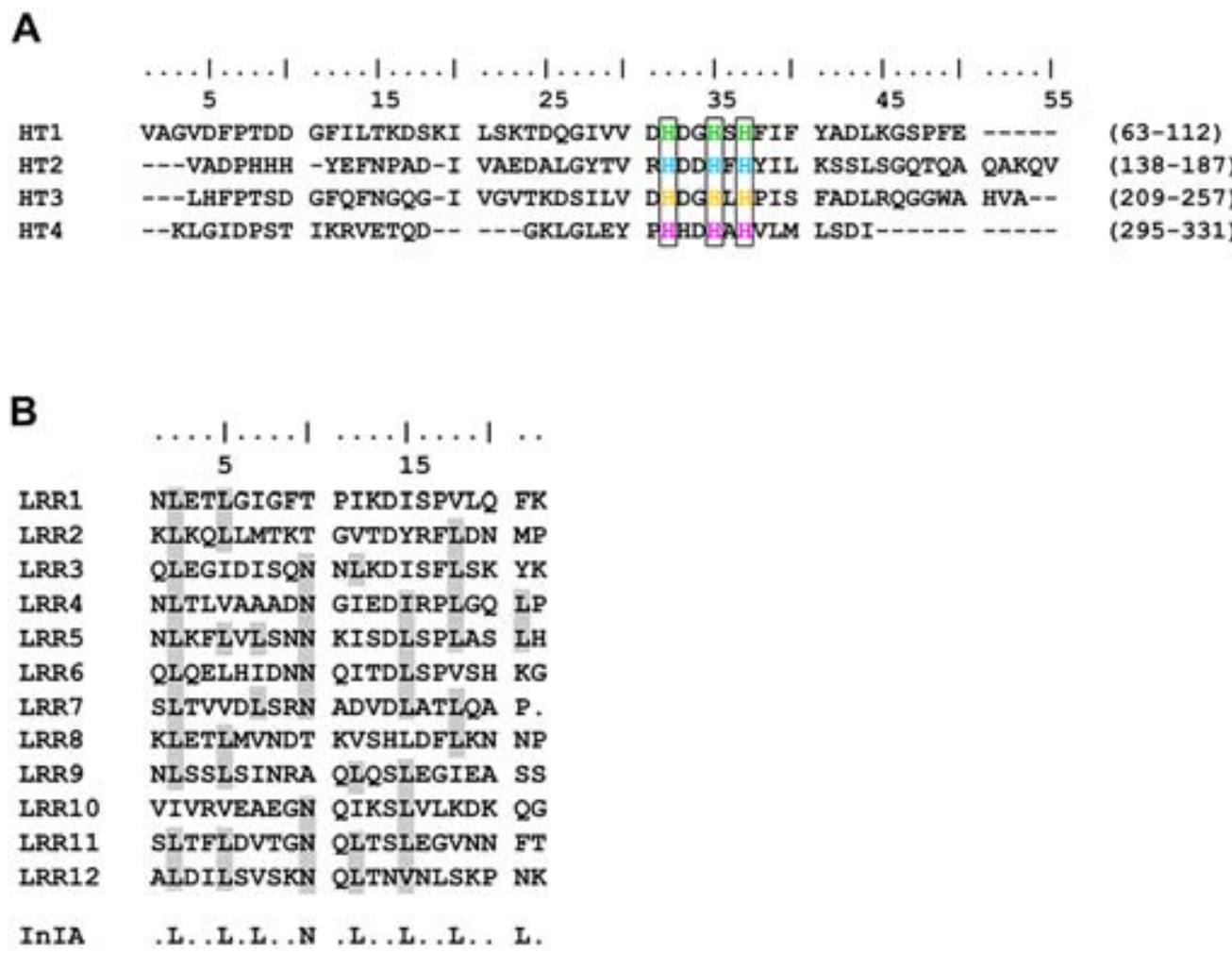

Figure EV4. Sequences of repeats in the HT and LRR regions of Slr.

(**A**) Alignment of sequences of the Histidine Triad (HT)1, HT2, HT3, and HT4 domains of Slr. The sequences shown are based on the predicted structural features of the HT regions. Each HT is between 37 and 50 amino acid residues. The Histidine Triad sequence is composed of the consensus sequence HXXHXH. The histidine residues in each HT region are colored. (**B**) Alignment of the 12 repeats in the LRR region. Each LRR has a length of 22 residues, except the seventh one, which is one residue shorter. The consensus sequence of LRRs in InlA of *L. monocytogenes* is indicated in bold below the alignment, and residues at the corresponding positions in Slr are highlighted in gray. Of note, all data in this figure are based on Slr in strain M5 Manfredo.
