# Supplementary material for: The Slr protein of Streptococcus pyogenes is selectively expressed in vivo and is a target for protective antibodies
Source: EMBO Mol Med. 2026 Jun 15;18(8):3086–108. doi: 10.1038/s44321-026-00470-0 (PMC13470328; doi:10.1038/s44321-026-00470-0)
Supplement: Supplementary file 2 [file 44321_2026_470_MOESM4_ESM.pdf]

EMBO Molecular Medicine

The Slr protein of Streptococcus pyogenes is selectively expressed in vivo and is a target for protective antibodies

Matevž Rumpret, Jonas Lannergård, Bodil Kristensen, Nicola Lynskey, Connor Bowen, Eskil Johnsson, Olof Nilsson, Anna Norrby-Teglund, Margaretha Stålhammar-Carlemalm, Gunnar Lindahl, and Alex McCarthy

Corresponding authors: Alex McCarthy (a.mccarthy@imperial.ac.uk) , Gunnar Lindahl (gunnar.lindahl@ple.lth.se)

Review Timeline:

Submission Date:	16th Jan 26
Editorial Decision:	9th Feb 26
Revision Received:	15th May 26
Editorial Decision:	2nd Jun 26
Revision Received:	4th Jun 26
Accepted:	5th Jun 26

Editor: Zeljko Durdevic

Transaction Report:

(Note: With the exception of the correction of typographical or spelling errors that could be a source of ambiguity, letters and reports are not edited. Depending on transfer agreements, referee reports obtained elsewhere may or may not be included in this compilation. Referee reports are anonymous unless the Referee chooses to sign their reports.)

9th Feb 2026

Dear Dr. McCarthy,

Thank you for the submission of your manuscript to EMBO Molecular Medicine. We have now received feedback from the three reviewers who agreed to evaluate your manuscript. All three referees recognize interest of the study but also raise serious and partially overlapping concerns that should be addressed in a major revision. If you would like to discuss further the points raised by the referees, I am available to do so via email or video. Let me know if you are interested in this option.

Further consideration of a revision that addresses reviewers' concerns in full will entail a second round of review. EMBO Molecular Medicine encourages a single round of revision only and therefore, acceptance or rejection of the manuscript will depend on the completeness of your responses included in the next, final version of the manuscript. For this reason, and to save you from any frustrations in the end, I would strongly advise against returning an incomplete revision.

We would welcome the submission of a revised version within three months for further consideration. Please let us know if you require longer to complete the revision.

I look forward to receiving your revised manuscript.

Yours sincerely,

Zeljko Durdevic

Zeljko Durdevic
Editor
EMBO Molecular Medicine

*****
When preparing your revised manuscript, please refer to our guidelines: https://link.springer.com/journal/44321/submission-guidelines#cms-Revised-submissions. We perform an initial quality control of all revised manuscripts before re-review; failure to include requested items will delay the evaluation of your revision.

We require:

1) A .docx formatted version of the manuscript text (including legends for main figures, EV figures and tables). Please make sure that the changes are highlighted to be clearly visible.

2) Individual production quality figure files as .eps, .tif, .jpg (one file per figure). For guidance, download the 'Figure Guide PDF': https://media.springernature.com/original/springer-cms/rest/v1/content/27825798/data/v1.

3) A .docx formatted letter INCLUDING the reviewers' reports and your detailed point-by-point responses to their comments. As part of the EMBO Press transparent editorial process, the point-by-point response is part of the Review Process File (RPF), which will be published alongside your paper.

4) A complete author checklist, which you can download from our author guidelines. Please insert information in the checklist that is also reflected in the manuscript. The completed author checklist will also be part of the RPF.

5) Please note that all corresponding authors are required to supply an ORCID ID for their name upon submission of a revised manuscript.

6) It is mandatory to include a 'Data Availability' section after the Materials and Methods. Before submitting your revision, primary datasets produced in this study need to be deposited in an appropriate public database, and the accession numbers and

database listed under 'Data Availability'. Please remember to provide a reviewer password if the datasets are not yet public.

In case you have no data that requires deposition in a public database, please state so in this section. Note that the Data Availability Section is restricted to new primary data that are part of this study.

7) For data quantification: please specify the name of the statistical test used to generate error bars and P values, the number (n) of independent experiments (specify technical or biological replicates) underlying each data point and the test used to calculate p-values in each figure legend. The figure legends should contain a basic description of n, P and the test applied. Graphs must include a description of the bars and the error bars (s.d., s.e.m.).

8) At EMBO Press we ask authors to provide source data for the main manuscript figures. You will receive a separate email with instructions for providing source data with your revised manuscript, including how to upload and organize the files.

9) Our journal encourages inclusion of *data citations in the reference list* to directly cite datasets that were re-used and obtained from public databases. Data citations in the article text are distinct from normal bibliographical citations and should directly link to the database records from which the data can be accessed. In the main text, data citations are formatted as follows: "Data ref: Smith et al, 2001" or "Data ref: NCBI Sequence Read Archive PRJNA342805, 2017". In the Reference list, data citations must be labeled with "[DATASET]". A data reference must provide the database name, accession number/identifiers and a resolvable link to the landing page from which the data can be accessed at the end of the reference.

10) We replaced Supplementary Information with Expanded View (EV) Figures and Tables that are collapsible/expandable online. A maximum of 5 EV Figures can be typeset. EV Figures should be cited as 'Figure EV1, Figure EV2" etc... in the text and their respective legends should be included in the main text after the legends of regular figures.

- For the figures that you do NOT wish to display as Expanded View figures, they should be bundled together with their legends in a single PDF file called *Appendix*, which should start with a short Table of Content. Appendix figures should be referred to in the main text as: "Appendix Figure S1, Appendix Figure S2" etc.

- Additional Tables/Datasets should be labeled and referred to as Table EV1, Dataset EV1, etc. Legends have to be provided in a separate tab in case of .xls files. Alternatively, the legend can be supplied as a separate text file (README) and zipped together with the Table/Dataset file.

11) The paper explained: EMBO Molecular Medicine articles are accompanied by a summary of the articles to emphasize the major findings in the paper and their medical implications for the non-specialist reader. Please provide a draft summary of your article highlighting

- the medical issue you are addressing,

- the results obtained and

- their clinical impact.

This may be edited to ensure that readers understand the significance and context of the research. Please refer to any of our published articles for an example.

12) Author contributions: You will be asked to provide CRediT (Contributor Role Taxonomy) terms in the submission system. These replace a narrative author contribution section in the manuscript.

13) A Conflict of Interest statement should be provided in the main text.

14) Every published paper includes a 'Synopsis' to further enhance discoverability. Synopses are displayed on the journal webpage and are freely accessible to all readers. They include a short stand first (maximum of 300 characters, including space) as well as 2-5 one-sentences bullet points that summarizes the paper. Please write the bullet points to summarize the key NEW findings. They should be designed to be complementary to the abstract - i.e. not repeat the same text. We encourage inclusion of key acronyms and quantitative information (maximum of 30 words / bullet point). Please use the passive voice. Please attach these in a separate file or send them by email, we will incorporate them accordingly.

Please also suggest a striking image or visual abstract to illustrate your article as a PNG file 550 px wide x 300-600 px high.

15) Include a Reagents and Tools Table as part of the Methods section, which can be downloaded from our author guidelines.

EMBO Molecular Medicine has a "scooping protection" policy, whereby similar findings that are published by others during review or revision are not a criterion for rejection. Should you decide to submit a revised version, I do ask that you get in touch

after three months if you have not completed it, to update us on the status.

***** Reviewer's comments *****

Referee #1 (Comments on Novelty/Model System for Author):

This manuscript presents novel characterization of Slr as a conserved, protective antigen that could be used to treat infections by the important pathogen S. pyogenes. The authors show that the protrein is likely expressed in vivo despite being poorly detectable during in vitro growth. With additional experimental support, if feasible, or expanded discussion addressing the points raised in the remarks sent to the authors, the study would make a valuable contribution to the field. I believe the manuscript is suitable for publication after revision, and I encourage the authors to address the points I have raised in the remarks sent to them either experimentally or at least through careful discussion in the context of the existing literature where experiments are not feasible.

Referee #1 (Remarks for Author):

This manuscript investigates the Streptococcus pyogenes lipoprotein Slr, a conserved antigen present in all strains, and addresses the apparent discrepancy between its lack of detectable surface expression under standard laboratory conditions and its ability to elicit protective immunity in vivo. Using passive and active immunization approaches in mouse models of invasive infection, the authors demonstrate that antibodies directed against Slr confer significant protection against multiple M types. The study further shows that Slr expression is regulated by the zinc-responsive transcriptional regulator AdcR and that derepression of this pathway leads to detectable surface expression in an adcR-deficient strain grown in vitro. In addition, the authors provide immunological analysis showing that Slr elicits strong antibody responses during both murine and human infection, with a pronounced immunodominance of the histidine-triad (HT) region over the LRR-containing region. Together, these findings support the important concept that the expression of antigens relevant for protective immunity should be studied for surface expression in vivo rather than under standard in vitro growth conditions.
Overall, this is a carefully performed and conceptually interesting study that addresses a central question in bacterial immunology and vaccine design, namely whether surface expression during laboratory growth is a prerequisite for an antigen to induce protective immunity. The work is relevant, technically solid, and provides new information on Slr as a conserved and potentially protective GAS antigen. The manuscript will be of interest to investigators working on streptococcal pathogenesis, host-pathogen interactions, and rational vaccine development.
That said, there are a few major points that, in my opinion, would benefit from clarification, additional experimental support and a more cautious framing of conclusions.
Major points
1. The title might give the impression that in vivo surface expression alone of any antigen is sufficient to confer protective immunity against S. pyogens once that antigen is formulated in a vaccine. I am not totally sure that this general principle has been demostrated beyond any doubt in the literature concerning S. pyogenes or other bacteria. Because the study deals with a single antigen, Slr, it would be perhaps helpful the frame the title in an antigen-specific manner rather than generalize to all S. pyogenes antigens.
2. A central premise of the manuscript is that Slr is selectively expressed on the bacterial surface during infection. I acknowledge that the ΔadcR mutant, but not the wild type, is clearly shown to express Slr on the surface when grown in vitro, confirming regulatory control by AdcR, However, direct experimental evidence demonstrating surface expression of Slr, or increased expression of the coding mRNA in bacteria recovered from infected animals is not provided. All flow cytometric analyses of surface exposure are performed on bacteria grown in vitro, including the ΔadcR mutant. While the strong antibody responses observed in infected mice and humans, together with the AdcR regulatory data, is compatible with in vivo expression, this is not directly proven, nor is surface expression, in particular, directly analyzed. Direct analysis of surface expression on bacteria present in infected tissues by microscopy (e.g. immunofluorescence or immune histochemistry or or immunofluorescence flow cytometry analysis of peritoneal exudates after removal of the majority of eucariotic cells e.g. by lysis and differential centrifugation) would substantially strengthen the manuscript. If such experiments are not possible, a more explicit discussion of this limitation and of the inferential nature of the conclusion would be appropriate. A a more clear and complete presentation and discussion of the existing evidence from the literature concerning increased in vivo expression of Srl would also perhaps be useful.
3. While the authors demonstrate that antibodies to Slr can be partially protective, the mechanism underlying this protection remains unclear. The manuscript implicitly assumes that surface accessibility and antibody binding are sufficient, but it is not addressed whether Slr functions as a virulence factor whose activity might be neutralized by antibodies, or whether protection is mediated primarily through opsonophagocytic mechanisms. In this context, bacteria killing in human blood after addition of antibodies against the antigen under study is a time-honored and highly reliable technique. Although this is a technically simple assay, if the authors are for any reasons unable to perform these experiments, the should perhaps acknowledge this open question and discuss alternative possibilities.
4. Fourth, the analysis of immunodominance versus subdominance within Slr is one of the most interesting aspects of the study. The authors convincingly show that the antibody response during infection is heavily skewed toward the HT region, while the LRR region is subdominant. Indeed, recombinant HT and LRR fragments were successfully produced and used in binding and inhibition assays. Given the availability of these fragments, it should be easy to use them to assess their protective activity after

active immunization with the fragments. Indeed, immunization experiments using isolated HT and LRR fragments would provide more valuable insight into which region(s) mediate protection and would strongly support the discussion on immune diversion and vaccine design. If such experiments cannot be performed, it would be helpful to even more explicitly frame the conclusions regarding protective epitopes as hypotheses rather than established findings.
Minor points
For completeness, the route of infection used in the mouse challenge experiments should be stated also in the materials and methods subsection: "Passive and active immunization of mice and subsequent infection", although it appears to be scattered in a few other points of the nanuscript.
First sentence, Abstract: "Vaccine candidates are commonly identified through characterization of microbial surface proteins expressed under laboratory conditions." "surface antigens" rather than "proteins" would be preferable, since protective antigens can of course also be carbohydrates.

Referee #2 (Comments on Novelty/Model System for Author):

The advance over what is known on Slr antigenicity, exposure, and regulation is relatively modest, and do not support that it is a particularly good vaccine target relative to all the others in progress.

Referee #2 (Remarks for Author):

There is no vaccine for strep A, a relatively common pathogen. Many antigenic targets have been examined, and some alone or in combination are in various stages of development. This work presents Slr, a conserved lipoprotein, as a viable target. Using some molecular techniques, the authors resolve some conflicting prior studies by showing it is not expressed well in culture, potentially making it an underappreciated target. They show some measure of protection for passive and active immunization in animal infection models, and that antibodies against it are formed during human infection. The study is overall well designed, though many caveats are presented in the following major points. However, it does overall present a relatively incremental advance - Slr regulation already known, that its is antigenic is known, and that antibodies against it can be protective is known.

In no animal experiments is information on the bacterial challenge given in text, legend, or methods - what kind of infection model is this? It cannot be evaluated if whether this is an appropriate test or the conclusions justified based on this.

The premise states that Slr must be on the cell surface during infections, such as in mice, though it absent in vitro. This should be shown. Performing FACS on strep from infected mice, can slr be detected on the cell surface similar to M, as claimed?

The focus of aim 2 is somewhat bizarre in its justification, why examine Slr as a surface protein if it's not expressed anyway? Nonetheless, if analyzing it by antibody, there should be positive controls using that antibody by FACS. If other factors limit exposure of Slr (Waldemarsson et al.2006), this should be detailed further. However, this point shouldn't misconstrue the points on its exposure as an antigen vs expression.

The question is only partially addressed using recombinant protein in Fig 4C; whether anti-slr antibodies contribute significantly would be better addressed with cell lysates, including from slr and emm mutant controls, examining for strength of the anti-Slr band and its disappearance in the lane for the mutant strain. As is, the blots are not convincing

Controls of serum from healthy humans and mice are important for the interpretation of Fig 4.

Does not offer sufficient discussion on differing conclusions from prior studies (Bensi et al. 2012; Fritzer et al. 2010) in statements like "Contrary to our observations, two previous studies reported that active immunization with Slr did not confer protection (Fritzer et al. 2010), or protected against only one of three S. pyogenes strains (Bensi et al. 2012). The various results cannot be directly compared, but our results now indicate that Slr is of interest as a component of a future S. pyogenes vaccine." are not well-developed scholarship - how is this work placed in the field and what insights can be taken from these studies toward the natural human condition?

Additional discussion on the points that Slr present in other ubiquitous streptococci, whether healthy humans have pre-existing antibodies, that these are cross-reactive between species, and that these are not protective, are warranted. Further, we know humans get recurrent infections, though this is potentially added by antigenic escpate mediated by M protein. Wouldn't this suggest anti-Slr antibodies aren't naturally protective?

The IVET discussion (lines 366-372) is odd and out of date; it's a fine technique, but for the stated purpose of examining "upregulation in vivo", RNAseq or proteomics is what nearly any modern study would perform, and has been done, yet has no

discussion

Referee #3 (Comments on Novelty/Model System for Author):

The manuscript by Rumpret et al. is a clear and meticulous study, investigating the role of Slr as a vaccine antigen. It addresses several important concepts for vaccine development, including in-vivo expressed antigens not produced under standard lab conditions, and the concept of immunodominant and subdominant (cryptic?) protective epitopes.

Comments for the authors consideration:

Line 126: State which adjuvant used for this study here.

Line 146-162: I commend the authors for clearing up these contradictory previous publications.

Lines 236-238: Cannot compare the immune response between M protein and Slr, because the coating antigen is different e.g. may bind at different levels to the ELISA plate. Unless you wish to calculate out in terms of e.g. ug specific IgG/ml?

Lines 253-310. The immunodominant HT which is non-protective vs subdominant LRR is an attractive hypothesis. Have the authors tested this hypothesis using either passive or active immunisation?

Lines 333-334: Can the authors state which adjuvants were used in the Fritzer and Bensi studies? This would be a useful comparator to this current work.

Figure 1D-G: Did the authors monitor beyond 96 h? Wass the level of efficacy maintained in the Slr group and M protein positive control group?

Referee #3 (Remarks for Author):

Overall, this manuscript makes a strong contribution to the field.

I would like to see the authors test the hypothesis that the immunodominant HT which is non-protective vs subdominant LRR is an attractive hypothesis, using either passive or active immunisation?

Referee #1 (Comments on Novelty/Model System for Author):

This manuscript presents novel characterization of Slr as a conserved, protective antigen that could be used to treat infections by the important pathogen S. pyogenes. The authors show that the protrein is likely expressed in vivo despite being poorly detectable during in vitro growth. With additional experimental support, if feasible, or expanded discussion addressing the points raised in the remarks sent to the authors, the study would make a valuable contribution to the field. I believe the manuscript is suitable for publication after revision, and I encourage the authors to address the points I have raised in the remarks sent to them either experimentally or at least through careful discussion in the context of the existing literature where experiments are not feasible.

Response: We thank the referee for this highly encouraging and supportive assessment of our manuscript. We appreciate the comments raised and have addressed each below. We note that the reviewer has requested the some points could be addressed by additional experiments or alternatively by textual revisions. We have included 1) new experimental data that investigated the functionality of anti-Slr IgGs in whole blood killing assays, as suggested by the reviewer, and 2) increased synthesis and discussion of data from our study and the literature.

Referee #1 (Remarks for Author):

This manuscript investigates the Streptococcus pyogenes lipoprotein Slr, a conserved antigen present in all strains, and addresses the apparent discrepancy between its lack of detectable surface expression under standard laboratory conditions and its ability to elicit protective immunity in vivo. Using passive and active immunization approaches in mouse models of invasive infection, the authors demonstrate that antibodies directed against Slr confer significant protection against multiple M types. The study further shows that Slr expression is regulated by the zinc-responsive transcriptional regulator AdcR and that derepression of this pathway leads to detectable surface expression in an adcR-deficient strain grown in vitro. In addition, the authors provide immunological analysis showing that Slr elicits strong antibody responses during both murine and human infection, with a pronounced immunodominance of the histidine-triad (HT) region over the LRR-containing region. Together, these findings support the important concept that the expression of antigens relevant for protective immunity should be studied for surface expression in vivo rather than under standard in vitro growth conditions.
Overall, this is a carefully performed and conceptually interesting study that addresses a central question in bacterial immunology and vaccine design, namely whether surface expression during laboratory growth is a prerequisite for an antigen to induce protective immunity. The work is relevant, technically solid, and provides new information on Slr as a conserved and potentially protective GAS antigen. The manuscript will be of interest to investigators working on streptococcal pathogenesis, host-pathogen interactions, and rational vaccine development.
That said, there are a few major points that, in my opinion, would benefit from clarification, additional experimental support and a more cautious framing of conclusions.

Response: We thank the referee for this positive summary of our findings.

Major points

1. The title might give the impression that in vivo surface expression alone of any antigen is sufficient to confer protective immunity against S. pyogens once that antigen is formulated in a vaccine. I am not totally sure that this general principle has been demostrated beyond any doubt in the literature concerning S. pyogenes or other bacteria. Because the study deals with a single antigen, Slr, it would be perhaps helpful the frame the title in an antigen-specific manner rather than generalize to all S. pyogenes antigens.

Response: We agree with the referee. We have modified the title to "The Slr protein of *Streptococcus pyogenes* is selectively expressed *in vivo* and is a target for protective antibodies"

2. A central premise of the manuscript is that Slr is selectively expressed on the bacterial surface during infection. I acknowledge that the ΔadcR mutant, but not the wild type, is clearly shown to express Slr on the surface when grown in vitro, confirming regulatory control by AdcR, However, direct experimental evidence demonstrating surface expression of Slr, or increased expression of the coding mRNA in bacteria recovered from infected animals is not provided. All flow cytometric analyses of surface exposure are performed on bacteria grown in vitro, including the ΔadcR mutant. While the strong antibody responses observed in infected mice and humans, together with the AdcR regulatory data, is compatible with in vivo expression, this is not directly proven, nor is surface expression, in particular, directly analyzed. Direct analysis of surface expression on bacteria present in infected tissues by microscopy (e.g. immunofluorescence or immune histochemistry or or immunofluorescence flow cytometry analysis of peritoneal exudates after removal of the majority of eucariotic cells e.g. by lysis and differential centrifugation) would substantially strengthen the manuscript. If such experiments are not possible, a more explicit discussion of this limitation and of the inferential nature of the conclusion would be appropriate. A a more clear and complete presentation and discussion of the existing evidence from the literature concerning increased in vivo expression of Srl would also perhaps be useful.

Response: We thank the referee for this constructive comment. We agree that direct visualisation of Slr on the surface of *S. pyogenes* recovered from infection would be very valuable. However, use of flow cytometry or other methods for this type of analysis presents significant technical challenge that we have not yet been able to overcome. Given this situation, we agree with the reviewer that a more explicit discussion of the situation is appropriate. Accordingly, we have revised our discussion and framed our conclusions as being supported by the convergence of immunological, clinical and transcription data. In particular, we have included a clearer presentation and discussion of the existing evidence from the literature concerning the increased in vivo expression of Slr, as requested. This text is now included at lines 370 to 386 in the Discussion:

“Despite the absence of Slr from the surface of laboratory-cultivated *S. pyogenes* strains, we found that it is a target for protective antibodies in infected mice, indicating that Slr is upregulated *in vivo*. Support for upregulation *in vivo* is provided by the demonstration that antibodies against Slr were elicited in *S. pyogenes*-infected macaques (Virtaneva *et al.*, 2003) and by our analysis of serum samples from infected humans and mice, indicating that Slr elicits an antibody response comparable to that elicited by M protein. Additionally, transcription studies show that transcription of the *slr* gene occurs at very low-level during growth in broth but is upregulated *in vivo* (Brenot *et al.*, 2007; Hirose *et al*, 2019; Kachroo *et al.*, 2020; Maamary *et al.*, 2012b; Makthal *et al.*, 2020; Sanson *et al.*, 2015). For instance, *slr* was found to be among the most highly expressed genes in *S. pyogenes* clinical isolates recovered from patients with either pharyngeal or invasive disease (Sumby *et al*, 2006). Furthermore, RNA-seq experiments have identified *slr* as being significantly upregulated during skin infection in a mouse model (Wilkening *et al*, 2023). While transcription data must be interpreted with caution, since the presence of a transcript *in vivo* does not necessarily imply that the protein is translated (Freiberg *et al*, 2016), all available data now support the notion that the Slr protein is virtually absent from laboratory-cultivated bacteria but is upregulated during infection”

3. While the authors demonstrate that antibodies to Slr can be partially protective, the mechanism underlying this protection remains unclear. The manuscript implicitly assumes that surface accessibility and antibody binding are sufficient, but it is not addressed whether Slr functions as a virulence factor whose activity might be neutralized by antibodies, or whether protection is mediated primarily through opsonophagocytic mechanisms. In this context, bacteria killing in human blood after addition of antibodies against the antigen under study is a time-honored and highly reliable technique. Although this is a technically simple assay, if the authors are for any reasons unable to perform these experiments, the should perhaps acknowledge this open question and discuss alternative possibilities.

Response: We thank the referee for this comment and agree that it is an important question to address. To assess this, we have measured the survival of *S. pyogenes* wildtype and Δ*adcR* in human blood following supplementation with rabbit preimmune sera (negative control), rabbit anti-M5 sera (positive control) or rabbit anti-Slr sera. These experiments revealed that the anti-Slr serum did not promote opsonophagocytic killing of the Δ*adcR* strain. This indicates that the anti-Slr antibodies do not protect by promoting opsonophagocytic uptake but likely neutralise the function of Slr. This new analysis is presented in a new Figure 3E, described on lines 602-608 in Methods and described as follows on lines 232-243 in Results:

“The demonstration that Slr is expressed on the surface of the *adcR* mutant made it possible to initiate an analysis of the role of functionality of anti-Slr antibodies. A major effector

mechanism of antibodies targeting surface-exposed *S. pyogenes* antigens is the facilitation of phagocytic uptake and killing by neutrophils in human whole blood through opsonisation. Specifically, we analyzed whether rabbit anti-Slr antibodies, which are protective in a mouse model (**Figure 1**), would opsonize the *adcR* mutant for phagocytosis in the classical bactericidal test employing human whole blood (**Figure 3E**). As expected, opsonisation with either anti-M5 or anti-M5-HVR serum resulted in a significant reduction in survival of Manfredo wild-type and D*adcR* strains. In contrast, opsonisation with anti-Slr IgG did not reduce outgrowth of Manfredo wildtype, as expected, or D*adcR* strains relative to the preimmune control. These data support the notion that antibodies to Slr primarily act by interfering with its function, not by promoting phagocytosis."

4. Fourth, the analysis of immunodominance versus subdominance within Slr is one of the most interesting aspects of the study. The authors convincingly show that the antibody response during infection is heavily skewed toward the HT region, while the LRR region is subdominant. Indeed, recombinant HT and LRR fragments were successfully produced and used in binding and inhibition assays. Given the availability of these fragments, it should be easy to use them to assess their protective activity after active immunization with the fragments. Indeed, immunization experiments using isolated HT and LRR fragments would provide more valuable insight into which region(s) mediate protection and would strongly support the discussion on immune diversion and vaccine design. If such experiments cannot be performed, it would be helpful to even more explicitly frame the conclusions regarding protective epitopes as hypotheses rather than established findings.

Response: We appreciate the referee's comments and agree that these experiments would be of interest. We note, however, that new immunisations and animal experiments, using isolated HT and LRR fragments, would be a large-scale investigation that is better suited to a future study. This important point is now mentioned in a new section on lines 437-451 in the Discussion, where we also consider the relevance of our findings for streptococcal species that express Slr-related proteins:

"Based on the arguments presented above, we propose a model, in which the antibody response to Slr during natural infection is directed towards the highly conserved HT region, allowing the subdominant LRR to evade antibodies and maintain function. This model is consistent with data on M proteins, for which the N-terminal hypervariable region (HVR) is subdominant but is a target for protective antibodies (Lannergård et al., 2011; Stalhammar-Carlemalm et al., 2007). We note that the specific protective efficacy of isolated LRR versus HT fragments needs to be formally analyzed in immunization and challenge studies. Regarding subdominance and protective immunity, it is of interest that Slr is the founding member of a family of streptococcal surface proteins with HT and LRR regions (Shao et al., 2013), implying that further studies of Slr may provide insights of relevance to the development of vaccines against other pathogens, including *Streptococcus agalactiae* and *Streptococcus dysgalactiae*. Since some of the Slr-like proteins cross-react (Waldemarsson et al., 2006), it is conceivable that antibodies to one Slr-like protein may protect against more

than one pathogen, providing an additional impetus for studies of these proteins as vaccine candidates."

Minor points

For completeness, the route of infection used in the mouse challenge experiments should be stated also in the materials and methods subsection: "Passive and active immunization of mice and subsequent infection", although it appears to be scattered in a few other points of the manuscript.

Response: We apologise for not providing this information clearly. We have updated Methods at lines 572 to 574 to indicate that mice were infected with *S. pyogenes* via the i.p. route. We have also added details to the relevant Figure legends.

First sentence, Abstract: "Vaccine candidates are commonly identified through characterization of microbial surface proteins expressed under laboratory conditions." "surface antigens" rather than "proteins" would be preferable, since protective antigens can of course also be carbohydrates.

Response: We agree, and we have updated, as requested.

Referee #2 (Comments on Novelty/Model System for Author):

The advance over what is known on Slr antigenicity, exposure, and regulation is relatively modest, and do not support that it is a particularly good vaccine target relative to all the others in progress.

Response: We respectfully disagree with this assessment. Contradicting previous work, our study demonstrates that Slr is not detectable on the bacterial surface under standard laboratory conditions but nevertheless is a target for protective antibodies during experimental infection. This finding focuses interest on the largely ignored concept that a vaccine antigen may be selectively expressed *in vivo*. As for vaccine development, it is noteworthy that no *S. pyogenes* vaccine is available, making it highly desirable to identify suitable candidates. Only future work can tell which candidate is particularly good.

Referee #2 (Remarks for Author):

There is no vaccine for strep A, a relatively common pathogen. Many antigenic targets have been examined, and some alone or in combination are in various stages of development. This work presents Slr, a conserved lipoprotein, as a viable target. Using some molecular techniques, the authors resolve some conflicting prior studies by showing it is not expressed well in culture, potentially making it an underappreciated target. They show some measure of protection for passive and active immunization in animal infection models, and that antibodies against it are formed during human infection. The study is overall well designed,

though many caveats are presented in the following major points. However, it does overall present a relatively incremental advance - Slr regulation already known, that its is antigenic is known, and that antibodies against it can be protective is known.

Response: We thank the referee for this assessment. We acknowledge that Slr has been previously identified as an antigen, but we respectfully disagree with the statement that our findings represent an incremental advance. We believe the significance of our work lies in the mechanistic synthesis of 3 lines that were poorly understood: (1) We unequivocally demonstrate that Slr is virtually absent from bacteria grown in the laboratory, contradicting data in the literature, (2) In contrast to earlier studies we clearly demonstrate that Slr is a target for protective antibodies during an infection, focusing interest on the paradigm that a key antibody target may be selectively expressed in vivo, and (3) We identify a hierarchy in the antibody response to different parts of Slr.

In no animal experiments is information on the bacterial challenge given in text, legend, or methods - what kind of infection model is this? It cannot be evaluated if whether this is an appropriate test or the conclusions justified based on this.

Response: We apologise for not providing this information clearly. We have updated the text in Methods at lines 572 to 574 to indicate that mice were infected with *S. pyogenes* via the i.p. route. We have also updated the relevant figure legends.

The premise states that Slr must be on the cell surface during infections, such as in mice, though it absent in vitro. This should be shown. Performing FACS on strep from infected mice, can slr be detected on the cell surface similar to M, as claimed?

Response: Please see response to Referee #1, Point 2.

The focus of aim 2 is somewhat bizarre in its justification, why examine Slr as a surface protein if it's not expressed anyway? Nonetheless, if analyzing it by antibody, there should be positive controls using that antibody by FACS. If other factors limit exposure of Slr (Waldemarsson et al.2006), this should be detailed further. However, this point shouldn't misconstrue the points on its exposure as an antigen vs expression.

Response: We thank the referee for this remark. In Figure 2, we establish that Slr is not detected on the surface of bacteria cultivated in the laboratory, and we support that observation by presenting gene expression data from publicly available datasets. By combining data on protein expression with transcription data, we avoid the problem that data on protein levels and mRNA levels commonly do not correlate well. As for positive controls in studies of surface expression, we used antibodies to M proteins, as extensively documented in the manuscript.

The question is only partially addressed using recombinant protein in Fig 4C; whether anti-slr antibodies contribute significantly would be better addressed with cell lysates, including from slr and emm mutant controls, examining for strength of the anti-Slr band and its disappearance in the lane for the mutant strain. As is, the blots are not convincing..

Response: We appreciate the referee's concern regarding the specificity of antibodies that detect Slr. However, it is not possible to analyse this problem by comparing lysates prepared from the wt and the *slr* mutant, as proposed by the referee, since Slr is not expressed by wt bacteria cultivated in the laboratory. On the other hand, Figure 3B shows analysis of lysates prepared from wt bacteria and the *adcR* mutant (which expresses Slr), and the results indicate that antibodies to Slr almost exclusively detect the Slr protein. The specificity of anti-Slr antibodies for Slr is also supported by the data in Figure 5, which show that human anti-Slr selectively reacts with the HT region of Slr. The specificity of the anti-Slr antibodies is now considered in a new sentence added to the section on antibody responses (lines 269-273):

"That the anti-Slr antibodies detected in this analysis indeed were elicited by Slr, and not by a cross-reacting antigen, is strongly supported by the Western blot analysis shown in Figure 3B, in which lysates prepared from wild type bacteria and the *adcR* mutant were compared, showing that anti-Slr mainly, if not exclusively, reacted with Slr."

Controls of serum from healthy humans and mice are important for the interpretation of Fig 4.

Response: We agree that robust controls are essential for interpreting serological data. For the studies of mouse sera, the legend of Figure 4 now mentions that normal mouse serum lacked reactivity in the tests. Regarding the tests with human sera, we utilised acute-phase sera as the primary control for convalescent-phase sera. This allows for the direct observation of seroconversion. Our data show a significant increase in anti-Slr and anti-M1 IgG titers from acute to convalescent phase, confirming that both Slr and M1 elicited antibody responses in the patients analyzed.

Does not offer sufficient discussion on differing conclusions from prior studies (Bensi et al. 2012; Fritzer et al. 2010) in statements like "Contrary to our observations, two previous studies reported that active immunization with Slr did not confer protection (Fritzer et al. 2010), or protected against only one of three S. pyogenes strains (Bensi et al. 2012). The various results cannot be directly compared, but our results now indicate that Slr is of interest as a component of a future S. pyogenes vaccine." are not well-developed scholarship - how is this work placed in the field and what insights can be taken from these studies toward the natural human condition?

Response: We thank the reviewer for this comment. We have revised our discussion at lines 355 to 363 as follows:-

"Our results contrast with previous studies in which Slr immunization did not confer protection (Fritzer *et al.*, 2010) or conferred protection against one of three *S. pyogenes* strains (Bensi *et al.*, 2012). These differences likely arise from differences in choice of challenge model (subcutaneous, intraperitoneal, intravenous) and adjuvant (which was Freund's or alum in the published report) (Bensi *et al.*, 2012; Fritzer *et al.*, 2010). More fundamentally, our data defining AdcR as a regulator suggests that Slr expression in *S.*

pyogenes is sensitive to environmental Zn. Therefore, differences in protection elicited by Slr immunization may also reflect different Zn levels and Slr expression by *S. pyogenes* in different tissue environments."

Additional discussion on the points that Slr present in other ubiquitous streptococci, whether healthy humans have pre-existing antibodies, that these are cross-reactive between species, and that these are not protective, are warranted. Further, we know humans get recurrent infections, though this is potentially added by antigenic escpate mediated by M protein. Wouldn't this suggest anti-Slr antibodies aren't naturally protective?

Response: We thank the reviewer for raising these points regarding natural immunity and cross-reactivity. For the present manuscript, it is particularly relevant that anti-Slr antibodies elicited during natural infection apparently are non-protective, although immunization with pure Slr elicits protective immunity, as shown in our paper. This problem was extensively discussed in the submitted version of our paper, a text included also in the revised version. As pointed out in that text, the apparent paradox may be explained by the subdominance of the LRR region in Slr. We have now extended the discussion of that model in the paragraph included below, which starts on line 437-451. (Of note, this paragraph was also included in the response to point 4 of Referee #1). As requested by the referee, this paragraph mentions that studies of Slr may provide information on the Slr-like proteins expressed by other streptococcal proteins, and we also mention that cross-reactivity among these proteins makes them of particular interest as vaccine candidates:

"Based on the arguments presented above, we propose a model, in which the antibody response to Slr during natural infection is directed towards the highly conserved HT region, allowing the subdominant LRR to evade antibodies and maintain function. This model is consistent with data on M proteins, for which the N-terminal hypervariable region (HVR) is subdominant but is a target for protective antibodies (Lannergård et al., 2011; Stalhammar-Carlemalm et al., 2007). We note that the specific protective efficacy of isolated LRR versus HT fragments needs to be formally analyzed in immunization and challenge studies. Regarding subdominance and protective immunity, it is of interest that Slr is the founding member of a family of streptococcal surface proteins with HT and LRR regions (Shao et al., 2013), implying that further studies of Slr may provide insights of relevance to the development of vaccines against other pathogens, including *Streptococcus agalactiae* and *Streptococcus dysgalactiae*. Since some of the Slr-like proteins cross-react (Waldemarsson et al., 2006), it is conceivable that antibodies to one Slr-like protein may protect against more than one pathogen, providing an additional impetus for studies of these proteins as vaccine candidates."

The IVET discussion (lines 366-372) is odd and out of date; it's a fine technique, but for the stated purpose of examining "upregulation in vivo", RNAseq or proteomics is what nearly any modern study would perform, and has been done, yet has no discussion.

Response: IVET was a foundational technique for identifying *in vivo*-induced genes. However, we agree that modern transcriptomic (RNA-seq) and proteomic approaches

provide a more contemporary view of the *S. pyogenes in vivo* landscape. We think that the two methods, IVET and RNA-seq, are complementary. We have therefore adjusted the discussion at lines 404 to 412:

"Our data, therefore, focus interest on transcriptomic profiling by RNA-seq and related methods, as well as complementary library screening approaches such as *in vivo* expression technology (IVET), which may together allow the identification of vaccine targets upregulated *in vivo* (Chiang *et al.*, 1999; Cummings & Relman, 2000; Lempke *et al*, 2023; Mahan *et al*, 1993a; Mahan *et al.*, 1993b; Rediers *et al*, 2005; Westermann & Vogel, 2021). While IVET has so far been used to a relatively limited extent, powerful high-resolution transcriptomic and proteomic approaches that have the capability to provide single-cell or spatial data have recently arisen, which may enable novel screening in streptococci to identify additional candidates for future vaccine development."

Referee #3 (Comments on Novelty/Model System for Author):

The manuscript by Rumpret et al. is a clear and meticulous study, investigating the role of Slr as a vaccine antigen. It addresses several important concepts for vaccine development, including in-vivo expressed antigens not produced under standard lab conditions, and the concept of immunodominant and subdominant (cryptic?) protective epitopes.

Response: We thank the referee for this highly encouraging and supportive assessment of our manuscript.

Comments for the authors consideration:

Line 126: State which adjuvant used for this study here.

Response: We have corrected as requested.

Line 146-162: I commend the authors for clearing up these contradictory previous publications.

Response: We appreciate the referee's comments.

Lines 236-238: Cannot compare the immune response between M protein and Slr, because the coating antigen is different e.g. may bind at different levels to the ELISA plate. Unless you wish to calculate out in terms of e.g. ug specific IgG/ml?

Response: We thank the referee for pointing out this technical aspect and we agree with the referee's comment. However, our intention was not to form a precise quantitative 1:1 equivalence of the anti-M and anti-Slr response, but to demonstrate that the anti-Slr response is a major component of the immune response during infection and that it is broadly comparable to the well-established anti-M response. To address the reviewer's

comment, we have revised the text in the Abstract (lines 38) and Results (line 267) to state "comparable".

Lines 253-310. The immunodominant HT which is non-protective vs subdominant LRR is an attractive hypothesis. Have the authors tested this hypothesis using either passive or active immunisation?

Response: We thank the referee for his/her interest in the hypothesis that antibodies to the immunodominant HT are non-protective. We have not tested this hypothesis. Please see the response to Reviewer 1, point 4.

Lines 333-334: Can the authors state which adjuvants were used in the Fritzer and Bensi studies? This would be a useful comparator to this current work.

Response: This is a valuable suggestion. We have incorporated the information as requested (lines 355-363).

Figure 1D-G: Did the authors monitor beyond 96 h? Wass the level of efficacy maintained in the Slr group and M protein positive control group?

Response: We did not monitor mouse survival in passive or active vaccinations beyond 96 hours, as 96 hours was the limit of ethical approval.

Referee #3 (Remarks for Author):

Overall, this manuscript makes a strong contribution to the field.

I would like to see the authors test the hypothesis that the immunodominant HT which is non-protective vs subdominant LRR is an attractive hypothesis, using either passive or active immunisation?

Response: Please see response to Reviewer 1 point 4.

2nd Jun 2026

Dear Dr. McCarthy,

Thank you for submitting your revised manuscript to EMBO Molecular Medicine. Your manuscript will be ready for acceptance pending the following final amendments:

1) Please address referee #2 minor point.
2) In the main manuscript file, please do the following:
- Please address all comments suggested by our data editors listed below:
o Figure legends:
1. Please note that the exact p values are not provided in the legends of figures 2B, 3C, E.
2. Please note that the box plots need to be defined in terms of minima, maxima, centre, bounds of box and whiskers, and percentile in the legend of figure 1E.
3. Please note that information related to n is missing in the legend of figure 4B.
4. Please note that the error bars are not defined in the legends of figures 4A, B.
- In Methods, provide the antibody dilutions that were used for each antibody.
- In Methods, provide the statement that the experiments involving human subjects conformed to the principles set out to the WMA Declaration of Helsinki and in the Department of Health and Human Services Belmont Report.
- Indicate in legends number and nature of replicates and exact p= values, not a range, along with the statistical test used. To keep the figures "clear" some authors found providing an Appendix table Sx with all exact p-values preferable. You are welcome to do this if you want to.
- Please rename "Conflict of interest information for all authors" to "Disclosure and competing interests statement". We updated our journal's competing interests policy in January 2022 and request authors to consider both actual and perceived competing interests. Please review the policy https://www.embopress.org/competing-interests and update your competing interests if necessary.
- Please remove the sentence "Has an open access license been selected: yes" from the conflict of interest statement.
- Author contributions: Please remove it from the manuscript and specify author contributions in our submission system. CRediT has replaced the traditional author contributions section because it offers a systematic machine-readable author contributions format that allows for more effective research assessment. You are encouraged to use the free text boxes beneath each contributing author's name to add specific details on the author's contribution. More information is available in our guide to authors:
https://www.embopress.org/page/journal/17574684/authorguide#authorshipguidelines
3) Funding: Make sure that information about all sources of funding are complete in both our submission system and in the manuscript. Currently Center for Nano-Vaccines, the Swedish Government Support for Clinical Research, the Trusts of Österlund and Golje, and the Royal Physiographic Society in Lund are missing in our submission system. Please correct.
4) Tables: Please remove Table EV1 and Table EV2 from the manuscript text and upload them as two separate files.
5) Source data: Please upload source data as one zipped file per figure. Fig 2A flow cytometry image can be removed. For the blot/gel images in Fig 3B and Fig 4C, the images should be annotated and uploaded as separate files (not pasted into an excel file).
6) The Paper Explained: The paper explained should be divided in 3 sections "Problem", "Results" and "Impact". Please check any of recently published EMBO Mol Med research articles for reference.
7) Synopsis:
- Please resize your synopsis image to exactly 550 pixels wide x 300 pixels high.
- Please check your synopsis text and image before submission with your revised manuscript. Please be aware that in the proof stage minor corrections only are allowed (e.g., typos).
8) As part of the EMBO Publications transparent editorial process (see our Editorial at http://embomolmed.embopress.org/content/2/9/329), EMBO Molecular Medicine will publish online a Review Process File (RPF) to accompany accepted manuscripts. This file will be published in conjunction with your paper and will include the anonymous referee reports, your point-by-point response and all pertinent correspondence relating to the manuscript. Let us know if you want to remove or not any figures from it prior to publication. Please note that the Authors checklist will be published at the end of the RPF.
9) Please provide a point-by-point letter INCLUDING my comments as well as the reviewer's reports and your detailed responses (as Word file).

I look forward to reading a new revised version of your manuscript as soon as possible.

Yours sincerely,

Zeljko Durdevic

Zeljko Durdevic
Senior Editor
EMBO Molecular Medicine

*** Instructions to submit your revised manuscript ***

*** PLEASE NOTE *** As part of the EMBO Publications transparent editorial process initiative (see our Editorial at https://www.embopress.org/doi/pdf/10.1002/emmm.201000094), EMBO Molecular Medicine will publish online a Review Process File to accompany accepted manuscripts.

In the event of acceptance, this file will be published in conjunction with your paper and will include the anonymous referee reports, your point-by-point response and all pertinent correspondence relating to the manuscript. If you do NOT want this file to be published, please inform the editorial office at contact@embomolmed.org.

*****

When preparing your revised manuscript, please refer to our guidelines: https://link.springer.com/journal/44321/submission-guidelines#cms-Revised-submissions. We perform an initial quality control of all revised manuscripts before re-review; failure to include requested items will delay the evaluation of your revision.

We require:

1) A .docx formatted version of the manuscript text (including legends for main figures, EV figures and tables). Please make sure that the changes are highlighted to be clearly visible.

2) Individual production quality figure files as .eps, .tif, .jpg (one file per figure). For guidance, download the 'Figure Guide PDF': https://media.springernature.com/original/springer-cms/rest/v1/content/27825798/data/v1.

3) A .docx formatted letter INCLUDING the reviewers' reports and your detailed point-by-point responses to their comments. As part of the EMBO Press transparent editorial process, the point-by-point response is part of the Review Process File (RPF), which will be published alongside your paper.

4) A complete author checklist, which you can download from our author guidelines. Please insert information in the checklist that is also reflected in the manuscript. The completed author checklist will also be part of the RPF.

5) Please note that all corresponding authors are required to supply an ORCID ID for their name upon submission of a revised manuscript.

6) It is mandatory to include a 'Data Availability' section after the Materials and Methods. Before submitting your revision, primary datasets produced in this study need to be deposited in an appropriate public database, and the accession numbers and database listed under 'Data Availability'. Please remember to provide a reviewer password if the datasets are not yet public.

In case you have no data that requires deposition in a public database, please state so in this section. Note that the Data Availability Section is restricted to new primary data that are part of this study.

7) For data quantification: please specify the name of the statistical test used to generate error bars and P values, the number (n) of independent experiments (specify technical or biological replicates) underlying each data point and the test used to calculate p-values in each figure legend. The figure legends should contain a basic description of n, P and the test applied. Graphs must include a description of the bars and the error bars (s.d., s.e.m.).

8) At EMBO Press we ask authors to provide source data for the main manuscript figures. You will receive a separate email with instructions for providing source data with your revised manuscript, including how to upload and organize the files.

9) Our journal encourages inclusion of *data citations in the reference list* to directly cite datasets that were re-used and obtained from public databases. Data citations in the article text are distinct from normal bibliographical citations and should

directly link to the database records from which the data can be accessed. In the main text, data citations are formatted as follows: "Data ref: Smith et al, 2001" or "Data ref: NCBI Sequence Read Archive PRJNA342805, 2017". In the Reference list, data citations must be labeled with "[DATASET]". A data reference must provide the database name, accession number/identifiers and a resolvable link to the landing page from which the data can be accessed at the end of the reference.

10) We replaced Supplementary Information with Expanded View (EV) Figures and Tables that are collapsible/expandable online. A maximum of 5 EV Figures can be typeset. EV Figures should be cited as 'Figure EV1, Figure EV2" etc... in the text and their respective legends should be included in the main text after the legends of regular figures.

- For the figures that you do NOT wish to display as Expanded View figures, they should be bundled together with their legends in a single PDF file called *Appendix*, which should start with a short Table of Content. Appendix figures should be referred to in the main text as: "Appendix Figure S1, Appendix Figure S2" etc.

- Additional Tables/Datasets should be labeled and referred to as Table EV1, Dataset EV1, etc. Legends have to be provided in a separate tab in case of .xls files. Alternatively, the legend can be supplied as a separate text file (README) and zipped together with the Table/Dataset file.

11) The paper explained: EMBO Molecular Medicine articles are accompanied by a summary of the articles to emphasize the major findings in the paper and their medical implications for the non-specialist reader. Please provide a draft summary of your article highlighting

- the medical issue you are addressing,

- the results obtained and

- their clinical impact.

This may be edited to ensure that readers understand the significance and context of the research. Please refer to any of our published articles for an example.

12) Author contributions: You will be asked to provide CRediT (Contributor Role Taxonomy) terms in the submission system. These replace a narrative author contribution section in the manuscript.

13) A Conflict of Interest statement should be provided in the main text.

14) Every published paper includes a 'Synopsis' to further enhance discoverability. Synopses are displayed on the journal webpage and are freely accessible to all readers. They include a short stand first (maximum of 300 characters, including space) as well as 2-5 one-sentences bullet points that summarizes the paper. Please write the bullet points to summarize the key NEW findings. They should be designed to be complementary to the abstract - i.e. not repeat the same text. We encourage inclusion of key acronyms and quantitative information (maximum of 30 words / bullet point). Please use the passive voice. Please attach these in a separate file or send them by email, we will incorporate them accordingly.

Please also suggest a striking image or visual abstract to illustrate your article as a PNG file 550 px wide x 300 px high.

15) Include a Reagents and Tools Table as part of the Methods section, which can be downloaded from our author guidelines.

EMBO Molecular Medicine has a "scooping protection" policy, whereby similar findings that are published by others during review or revision are not a criterion for rejection. Should you decide to submit a revised version, I do ask that you get in touch after three months if you have not completed it, to update us on the status.

*Additional important information regarding Figures

Each figure should be given in a separate file and should have the following resolution:
Graphs 800-1,200 DPI
Photos 400-800 DPI
Colour (only CMYK) 300-400 DPI"

Figures are not edited by the production team. All lettering should be the same size and style; figure panels should be indicated by capital letters (A, B, C etc). Gridlines are not allowed except for log plots. Figures should be numbered in the order of their appearance in the text with Arabic numerals. Each Figure must have a separate legend and a caption is needed for each panel.

*Additional important information regarding figures and illustrations can be found at
https://media.springernature.com/original/springer-cms/rest/v1/content/27825798/data/v1

***** Reviewer's comments *****

Referee #1 (Remarks for Author):

The authors have adequately addressed the points I have raised

Referee #2 (Remarks for Author):

The revision adequately clears up technical issues, and brings the claims more in line with the data. The additional experiments further strengthen the manuscript. Altogether, these support the basic premise of Slr as an antigen that is further advanced in this work. As a minor point, Figure EV1C is completely unclear, it possibly appears that there are regions of greater variability between strains, but the resultion, lack of labels, etc, make this unclear. Perhaps consolidating the redundancy or using another schema would help this

The authors addressed the remaining editorial issues.

5th Jun 2026

Dear Dr. McCarthy,

We are pleased to inform you that your manuscript is accepted for publication and is now being sent to our publisher to be included in the next available issue of EMBO Molecular Medicine.

Your manuscript will be processed for publication by EMBO Press. It will be copy edited and you will receive page proofs prior to publication. Please note that you will be contacted by Springer Nature Author Services to complete licensing and payment information.

You may qualify for financial assistance for your publication charges - either via a Springer Nature fully open access agreement or an EMBO initiative. Check your eligibility: https://link.springer.com/journal/44321/how-to-publish-with-us

Should you be planning a Press Release on your article, please notify the production team via this form in order to coordinate publication and release dates: https://forms.cloud.microsoft/e/eH4A8JVP6g

If you have any questions, please do not hesitate to contact the Editorial Office. Thank you for your contribution to EMBO Molecular Medicine.

Yours sincerely,
Zeljko Durdevic

Zeljko Durdevic
Senior Editor
EMBO Molecular Medicine

>>> Please note that it is EMBO Molecular Medicine policy for the transcript of the editorial process (containing referee reports and your response letter) to be published as an online supplement to each paper. If you do NOT want this, you will need to inform the Editorial Office via email immediately. More information is available here: https://link.springer.com/partners/embo-press/editorial-policies#Peer%20review

EMBO Press Author Checklist

Corresponding Author Name: Alex J. McCarthy	
Journal Submitted to: EMBO Molecular Medicine	
Manuscript Number: EMM-2026-23330-T	

USEFUL LINKS FOR COMPLETING THIS FORM
The EMBO Journal - Author Guidelines
EMBO Reports - Author Guidelines
Molecular Systems Biology - Author Guidelines
EMBO Molecular Medicine - Author Guidelines

Reporting Checklist for Life Science Articles (updated January
This checklist is adapted from Materials Design Analysis Reporting (MDAR) Checklist for Authors. MDAR establishes a minimum set of requirements in transparent reporting in the life sciences (see Statement of Task: 10.31222/osf.io/9sm4x). Please follow the journal's guidelines in preparing your
Please note that a copy of this checklist will be published alongside your article.

Abridged guidelines for figures
1. Data
The data shown in figures should satisfy the following conditions:
- the data were obtained and processed according to the field's best practice and are presented to reflect the results of the experiments in an accurate and unbiased manner.
- ideally, figure panels should include only measurements that are directly comparable to each other and obtained with the same assay.
- plots include clearly labeled error bars for independent experiments and sample sizes. Unless justified, error bars should not be shown for technical
- if n<5, the individual data points from each experiment should be plotted. Any statistical test employed should be justified.
- Source Data should be included to report the data underlying figures according to the guidelines set out in the authorship guidelines on Data

2. Captions
Each figure caption should contain the following information, for each panel where they are relevant:
- a specification of the experimental system investigated (eg cell line, species name).
- the assay(s) and method(s) used to carry out the reported observations and measurements.
- an explicit mention of the biological and chemical entity(ies) that are being measured.
- an explicit mention of the biological and chemical entity(ies) that are altered/varied/perturbed in a controlled manner.
- the exact sample size (n) for each experimental group/condition, given as a number, not a range;
- a description of the sample collection allowing the reader to understand whether the samples represent technical or biological replicates (including how many animals, litters, cultures, etc.).
- a statement of how many times the experiment shown was independently replicated in the laboratory.
- definitions of statistical methods and measures:
  - common tests, such as t-test (please specify whether paired vs. unpaired), simple χ2 tests, Wilcoxon and Mann-Whitney tests, can be unambiguously identified by name only, but more complex techniques should be described in the methods section;
  - are tests one-sided or two-sided?
  - are there adjustments for multiple comparisons?
  - exact statistical test results, e.g., P values = x but not P values < x;
  - definition of 'center values' as median or average;
  - definition of error bars as s.d. or s.e.m.

Please complete ALL of the questions below.
Select "Not Applicable" only when the requested information is not relevant for your study.

Materials

Newly Created Materials	Information included in the manuscript?	In which section is the information available? (Reagents and Tools Table, Materials and Methods, Figures, Data Availability Section)
New materials and reagents need to be available; do any restrictions apply?	Yes	Data Availability Section

Antibodies	Information included in the manuscript?	In which section is the information available? (Reagents and Tools Table, Materials and Methods, Figures, Data Availability Section)
For **antibodies** provide the following information:<br>- Commercial antibodies: RRID (if possible) or supplier name, catalogue number and or/clone number<br>- Non-commercial: RRID or citation	Yes	Material and Methods, Reagents and Tools Table

DNA and RNA sequences	Information included in the manuscript?	In which section is the information available? (Reagents and Tools Table, Materials and Methods, Figures, Data Availability Section)
**Short novel DNA or RNA including primers, probes**: provide the sequences.	Yes	Table EV2

Cell materials	Information included in the manuscript?	In which section is the information available? (Reagents and Tools Table, Materials and Methods, Figures, Data Availability Section)
**Cell lines:** Provide species information, strain. Provide accession number in repository **OR** supplier name, catalog number, clone number, and/**OR** RRID.	Not Applicable	
**Primary cultures:** Provide species, strain, sex of origin, genetic modification status.	Not Applicable	
Report if the cell lines were recently **authenticated** (e.g., by STR profiling) and tested for mycoplasma contamination.	Not Applicable	

Experimental animals	Information included in the manuscript?	In which section is the information available? (Reagents and Tools Table, Materials and Methods, Figures, Data Availability Section)
**Laboratory animals or Model organisms:** Provide species, strain, sex, age, genetic modification status. Provide accession number in repository **OR** supplier name, catalog number, clone number, **OR** RRID.	Yes	Reagents and Tools Table
**Animal observed in or captured from the field:** Provide species, sex, and age where possible.	Not Applicable	
Please detail **housing and husbandry conditions**.	Yes	Ethics Statement

Plants and microbes	Information included in the manuscript?	In which section is the information available? (Reagents and Tools Table, Materials and Methods, Figures, Data Availability Section)
**Plants:** provide species and strain, ecotype and cultivar where relevant, unique accession number if available, and source (including location for collected wild specimens).	Not Applicable	
**Microbes:** provide species and strain, unique accession number if available, and source.	Yes	Reagents and Tools Table, Table EV1

Human research participants	Information included in the manuscript?	In which section is the information available? (Reagents and Tools Table, Materials and Methods, Figures, Data Availability Section)
If collected and within the bounds of privacy constraints report on age, sex and gender or ethnicity for all study participants.	Yes	Reagents and Tools Table, Ethics Statement

Core facilities	Information included in the manuscript?	In which section is the information available? (Reagents and Tools Table, Materials and Methods, Figures, Data Availability Section)
If your work benefited from core facilities, was their service mentioned in the acknowledgments section?	Not Applicable	

Design

Study protocol	Information included in the manuscript?	In which section is the information available? (Reagents and Tools Table, Materials and Methods, Figures, Data Availability Section)
If study protocol has been **pre-registered, provide DOI in the manuscript**. For clinical trials, provide the trial registration number **OR** cite DOI.	Not Applicable	
Report the **clinical trial registration number** (at ClinicalTrials.gov or equivalent), where applicable.	Not Applicable	

Laboratory protocol	Information included in the manuscript?	In which section is the information available? (Reagents and Tools Table, Materials and Methods, Figures, Data Availability Section)
Provide DOI OR other citation details if **external detailed step-by-step protocols** are available.	Not Applicable	

Experimental study design and statistics	Information included in the manuscript?	In which section is the information available? (Reagents and Tools Table, Materials and Methods, Figures, Data Availability Section)
Include a statement about **sample size** estimate even if no statistical methods were used.	Yes	Materials and Methods, Figures
Were any steps taken to minimize the effects of subjective bias when allocating animals/samples to treatment (e.g. **randomization procedure**)? If yes, have they been described?	Not Applicable	
Include a statement about **blinding** even if no blinding was done.	Not Applicable	
Describe **inclusion/exclusion criteria** if samples or animals were excluded from the analysis. Were the criteria pre-established?<br><br>If sample or data points were omitted from analysis, report if this was due to attrition or intentional exclusion and provide justification.	Not Applicable	
For every figure, are **statistical tests** justified as appropriate? Do the data meet the assumptions of the tests (e.g., normal distribution)? Describe any methods used to assess it. Is there an estimate of variation within each group of data? Is the variance similar between the groups that are being statistically compared?	Yes	Materials and Methods, Figures

Sample definition and in-laboratory replication	Information included in the manuscript?	In which section is the information available? (Reagents and Tools Table, Materials and Methods, Figures, Data Availability Section)
In the figure legends: state number of times the experiment was **replicated** in laboratory.	Yes	Figures
In the figure legends: define whether data describe **technical or biological replicates**.	Yes	Figures

Ethics

Ethics	Information included in the manuscript?	In which section is the information available? (Reagents and Tools Table, Materials and Methods, Figures, Data Availability Section)
Studies involving **human participants**: State details of **authority granting ethics approval** (IRB or equivalent committee(s), provide reference number for approval.	Yes	Ethics Statement
Studies involving **human participants**: Include a statement confirming that **informed consent** was obtained from all subjects and that the experiments conformed to the principles set out in the WMA Declaration of Helsinki and the Department of Health and Human Services Belmont Report.	Yes	Ethics Statement
Studies involving **human participants: ** For publication of **patient photos**, include a statement confirming that consent to publish was obtained.	Not Applicable	
Studies involving experimental **animals**: State details of **authority granting ethics approval** (IRB or equivalent committee(s), provide reference number for approval. Include a statement of compliance with ethical regulations.	Yes	Ethics Statement
Studies involving **specimen and field samples:** State if relevant **permits** obtained, provide details of authority approving study; if none were required, explain why.	Not Applicable	

Dual Use Research of Concern (DURC)	Information included in the manuscript?	In which section is the information available? (Reagents and Tools Table, Materials and Methods, Figures, Data Availability Section)
Could your study fall under dual use research restrictions? Please check biosecurity documents and list of **select agents and toxins** (CDC): https://www.selectagents.gov/sat/list.htm	Not Applicable	
If you used a select agent, is the security level of the lab appropriate and reported in the manuscript?	Not Applicable	
If a study is subject to dual use research of concern regulations, is the name of the **authority granting approval and reference number** for the regulatory approval provided in the manuscript?	Not Applicable	

Reporting

The MDAR framework recommends adoption of discipline-specific guidelines, established and endorsed through community initiatives. Journals have their own policy about requiring specific guidelines and recommendations to complement MDAR.

Adherence to community standards	Information included in the manuscript?	In which section is the information available? (Reagents and Tools Table, Materials and Methods, Figures, Data Availability Section)
State if relevant guidelines or checklists (e.g., **ICMJE, MIBBI, ARRIVE, PRISMA**) have been followed or provided.	Not Applicable	
For **tumor marker prognostic studies,** we recommend that you follow the **REMARK** reporting guidelines (see link list at top right). See author guidelines, under 'Reporting Guidelines'. Please confirm you have followed these guidelines.	Not Applicable	
For **phase II and III randomized controlled trials**, please refer to the **CONSORT** flow diagram (see link list at top right) and submit the CONSORT checklist (see link list at top right) with your submission. See author guidelines, under 'Reporting Guidelines'. Please confirm you have submitted this list.	Not Applicable	

Data Availability

Data availability	Information included in the manuscript?	In which section is the information available? (Reagents and Tools Table, Materials and Methods, Figures, Data Availability Section)
Have **primary datasets** been deposited according to the journal's guidelines (see 'Data Deposition' section) and the respective accession numbers provided in the Data Availability Section?	Not Applicable	
Were **human clinical and genomic datasets** deposited in a public access-controlled repository in accordance to ethical obligations to the patients and to the applicable consent agreement?	Not Applicable	
Are **computational models** that are central and integral to a study available without restrictions in a machine-readable form? Were the relevant accession numbers or links provided?	Not Applicable	
If publicly available data were reused, provide the respective **data citations in the reference list**.	Yes	Reference list
